# Interactions between long- and short-term synaptic plasticity transform temporal neural representations into spatial

**DOI:** 10.1073/pnas.2426290122

**Published:** 2025-11-21

**Authors:** Qiang Yu, Misha Tsodyks, Haim Sompolinsky, Dietmar Schmitz, Robert Gütig

**Affiliations:** ^a^School of Artificial Intelligence, Tianjin Key Laboratory of Cognitive Computing and Application, College of Intelligence and Computing, Tianjin University, Tianjin 300350, China; ^b^Department of Brain Sciences, Weizmann Institute of Science, Rehovot 76100, Israel; ^c^School of Natural Sciences, Institute for Advanced Study, Princeton, NJ 08540; ^d^Center for Brain Science, Harvard University, Cambridge, MA 02138; ^e^Edmond and Lily Safra Center for Brain Sciences, Hebrew University of Jerusalem, Jerusalem 9190401, Israel; ^f^Neuroscience Research Center, Charité-Universitätsmedizin Berlin, Berlin 10117, Germany; ^g^Medical and Health Data Sciences, Berlin Institute of Health, Charité, Berlin, Germany

**Keywords:** short-term plasticity, long-term plasticity, supervised learning, storage capacity, spiking neurons

## Abstract

Information processing in the brain relies on the transmission of spikes through chemical synapses whose efficacies often depend on their recent firing history. While effects of such short-term plasticity on neural information processing have long been studied, it is unclear how interactions between short-term and long-term plasticity affect the learning abilities of neural networks. Here, we show that long-term changes to the short-term plasticity of individual synaptic connections enable neurons to learn to process temporal sequences of spikes as if they were spatial patterns. This mechanism allows neural circuits to flexibly increase their capacity and robustness at the expense of elevated spiking activity. We further show that neurons with plastic short-term plasticity can learn to discriminate between inputs on the basis of multineuronal spike correlations that extend over space and time. Our model fits recent electrophysiological measurements of short-term plasticity in the mouse and human neocortex and is consistent with the distributions of short-term plasticity induction and recovery. Our theory predicts that the learning rule observed at a given synaptic connection depends on the degree and type of short-term plasticity which is induced by the induction protocol for long-term plasticity.

## Significance

Neurons in the brain communicate through spikes that are transmitted via chemical synapses that express both long-term and short-term plasticity. While long-term plasticity is thought to be the central site of learning and memory and has dominated research, its interactions with short-term plasticity, i.e. the dynamics of the molecular transmission machinery, have largely remained unexplored. Using computational models that capture recent electrophysiological data from the mouse and human neocortex, we show that such interactions can greatly affect the learning abilities of neural networks and enable neurons to learn to process temporal sequences of spikes as if they were spatial patterns. This mechanism allows neural circuits to flexibly increase their capacity and robustness by increasing their activity instead of their size.

A hallmark of central nervous systems is the high degree of interconnectivity between neurons that typically connect to thousands of pre- and postsynaptic partners. In fact, the number of afferents that an individual neuron receives has long been established as a major determinant of its computational capabilities (1). However, wiring is expensive, both, because of the limited space within an animal’s skull and because of the associated maintenance costs (2). Therefore, it is important to understand to what extent, in addition to space, also the temporal dynamics of neural activity can enhance the computational capabilities of a given neural circuit. Because neurons communicate through spikes, this question translates into understanding how the number and temporal sequence of spikes can affect neural information processing.

Recently, the computational capabilities of spiking neural networks have been studied on the basis of supervised learning rules (3–11). However, these studies have assumed a static view with respect to synaptic transmission. In this view, synaptic efficacies are characterized through continuous scalar numbers that change only on slow time scales of learning across trials but are static and indifferent to the recent activity of a synapse, on the fast time scale of neural information processing. In contrast, signal transduction at chemical synapses in the brain results from complex molecular interactions between multiple biochemical processes whose dynamics result in substantial short-term plasticity of most connections: In the brain, synaptic efficacies often change from one spike to the next and these changes are typically sensitive to a synapse’s past activity on time scales of hundreds of milliseconds (12–15). The potential importance of short-term plasticity for a broad range of neural information processing tasks has long been recognized (15–18). These include temporal filtering (19–22), gain control (23), memory storage (24–26), information maximization (27, 28), as well as network stability and dynamics (29–37) and the multiplexing of feedforward and feedback signals (38). However, the majority of studies have been based on models of populations of synapses whose short-term plasticity was homogeneous and fixed. In contrast, short-term plasticity in the brain is highly diverse from connection to connection (29, 39–41) even when originating from the same presynaptic neuron (42) and can change when individual synapses undergo long-term plasticity (29, 42–44). While the idea that individual synapses learn their own short-term plasticity has long been proposed (45), its explorations have been scarce and lack generality. Specifically, previous studies were limited to nonspiking neural networks (46), discrete two-time-step models (47), heuristic population mean-field analyses (48), or were restricted to changes in release probabilities (49, 50). By contrast, the present work derives general gradient-based learning rules for spiking neurons with plastic short-term plasticity: two idealized forms that serve as reference and one well established phenomenological model that captures short-term plasticity in cortical circuits. We use these learning rules to study how plastic short-term plasticity of individual synaptic connections enhances spike-based neural information processing.

## Results

### From Single to Multiple Spikes per Afferent.

**Fig. 1.**
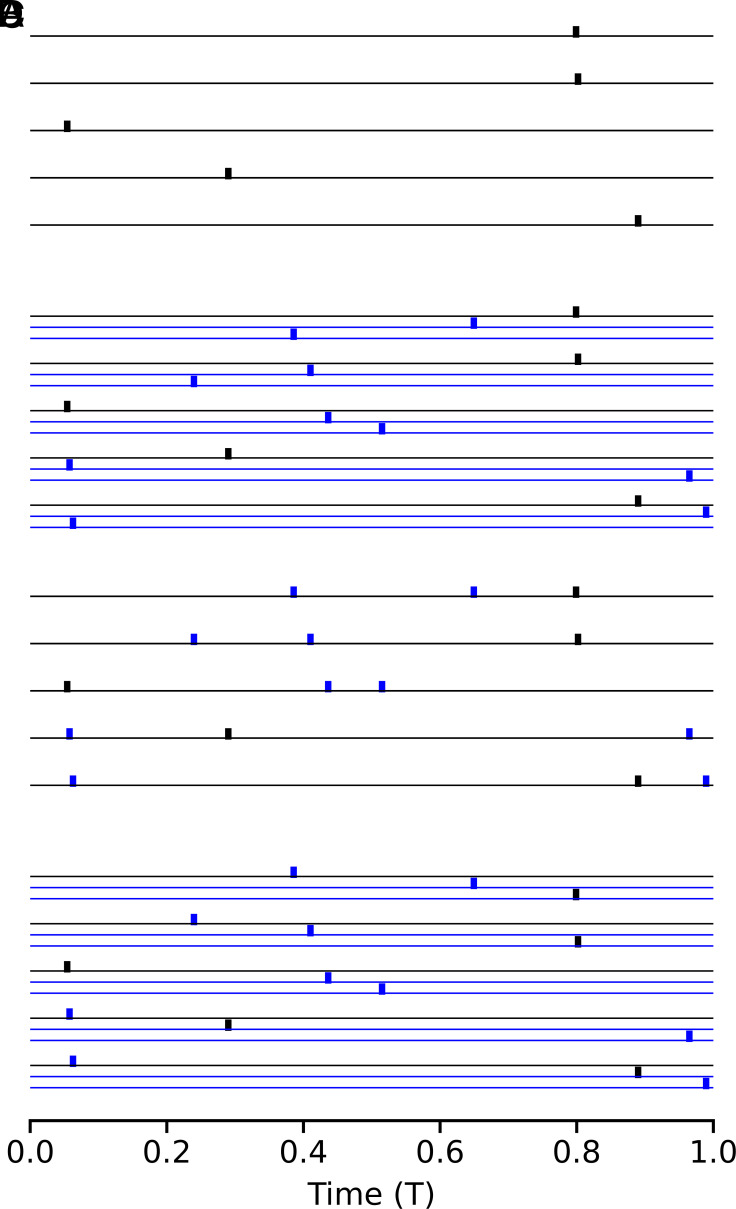
Input patterns. (*A*) Schematic example of a single-spike-per-afferent input pattern with each of the 5 afferents eliciting exactly one spike, at randomly drawn times, within the input interval from 0 to T. (*B*) Single-spike-per-afferent pattern as in (*A*) (black), however, with the number of afferents and spikes scaled by a factor of m=3, such that each afferent turns into a group of three. The 10 new afferents and spikes are shown in blue. (*C*) Example of a multiple-spikes-per-afferent pattern with the same m×N=15 spikes as in (*B*) but merged onto the initial N=5 afferents (c.f. panel *A*) such that each afferent now fires $n_{\mathrm{spikes}}=3$ spikes within the input interval. (*D*) Single-spike-per-afferent pattern as in (*B*) but with the temporal order constraint in place (see text for details) such that the spike times within each of the N=5 groups of 3 afferents are ordered in time.

Some progress toward understanding neural information processing in spiking neural networks has been achieved on the basis of supervised learning rules that have allowed for measurements of storage capacity (3, 5, 6). Using single-spike-per-afferent input patterns in which each afferent of a neuron fires exactly one spike within a given time interval (Fig. 1*A*), it has been observed (3) that the number of activity patterns that a neuron can learn to classify scales approximately linearly with the number of afferents. Such extensive scaling is well known from rate based neural networks, such as the perceptron, and is reflected by the important notions of learning load and storage capacity, which are defined relative to the number of afferents N. The load

[1] $$\begin{array}{c} \alpha =\frac{p}{N} \end{array}$$

refers to the number of patterns p per afferent that a neuron is required to classify in a given classification task. The storage capacity $\alpha_{c}$ designates the critical load for which the probability that a task can be realized by a given neural network is one half (1). Following previous studies (3, 5, 47), we will use storage capacity as a measure for the computational complexity of a given neural architecture.

Consider a thought experiment where we start out with a neuron with N afferents that can store p single-spike-per-afferent input patterns. Now we increase the number of afferents by a factor of m from N to N×m. For example, for m=3, we can think of each afferent splitting into 3 separate ones, e.g. afferent No. 1 turning into afferents No. 1-1, No. 1-2, and No. 1-3, afferent No. 2 turning into afferents No. 2-1, No. 2-2, and No. 2-3 and so on (Fig. 1*B*). In the one-spike-per-afferent scenario this means, that the total number of spikes per input pattern also grows from N to N×m. Importantly, following the above observation of extensive scaling in neurons with static synapses (3), the number of patterns that the neuron can now store grows by the same factor of m from p to p×m. In the above example with m=3, the neuron could store three times more patterns after each of its afferents turned into three afferents. Now imagine that we take the same sets of N×m spikes that make up the single-spike-per-afferent input patterns but keep the number of afferents fixed at N, so that now each afferent fires $n_{\mathrm{spikes}}=m$ spikes per pattern. In our example, the single spikes fired by afferents No. 1-1, No. 1-2, and No. 1-3 would now merge and form a sequence of three spikes on afferent No. 1 (Fig. 1*C*). How many of these multiple-spikes-per-afferent patterns can the neuron learn to classify? Will it be more than the initial p patterns, perhaps because the additional spikes make the patterns more distinguishable from each other?

### Idealized Short-Term Plasticity: Ordinal Synapses.

Before checking this multiple-spikes-per-afferent scenario for a neuron with static synapses, let us think about what would happen if the neuron’s synapses were dynamic, i.e. would express short-term plasticity, such that instead of weighting each of the $n_{\mathrm{spikes}}$ spikes of a given afferent with the same static efficacy, the efficacy could change from one spike to the next. In the brain, the efficacies of a synapse are complicated functions of its recent activity history and constrained by the type of dynamics that govern the molecular transmission machinery. However, let us first consider the limiting case of a synapse whose short-term plasticity we imagine to be so rich that each of its efficacies for successive spikes can be chosen independently. Specifically, we can characterize this synapse by $n_{\mathrm{spikes}}$ independent efficacies that correspond to its strengths for each successive activation, i.e. one for the first spike, one for the second spike, and so forth. We call this idealized synapse the ordinal synapse because its efficacy is determined by the rank order of the spikes within a sequence but does not depend on the specific intervals between them (*Materials and Methods*: Ordinal synapse, Eq. **7**). In the ordinal synapse case, we can interpret each of the N afferents that receive a sequence of $n_{\mathrm{spikes}}$ spikes from a multiple-spikes-per-afferent input pattern as a group of $n_{\mathrm{spikes}}$ afferents with static synapses that receive a single-spike-per-afferent input pattern. In our above example, we could interpret afferent No. 1 with one ordinal synapse as the group of afferents No. 1-1, No. 1-2, and No. 1-3 with three static synapses. Importantly, this projection of temporal sequences of spikes to spatial patterns of spikes entails that the order of spikes within the spatial patterns is fixed, mimicking the sequential activation of a dynamic synapse. Specifically, in the example, the first spike of the sequence of three spikes arriving on afferent No. 1 with the ordinal synapse (Fig. 1*C*) would always be assigned to afferent No. 1-1 with a static synapse, the second spike to afferent No. 1-2 and the third to afferent No. 1-3 (Fig. 1*D*). As a result, the multiple-spikes-per-afferent scenario with ordinal synapses is equivalent to the expanded single-spike-per-afferent scenario with static synapses if all input patterns are constrained such that the order of spikes within each group of $n_{\mathrm{spikes}}$ afferents is fixed. We use the capacity of the neuron model in this ordinal synapse scenario as a reference, because it is unconstrained by the biophysical limitations of the synaptic transmission machinery.

### A Fundamental Limit of Static Synaptic Transmission.

**Fig. 2.**
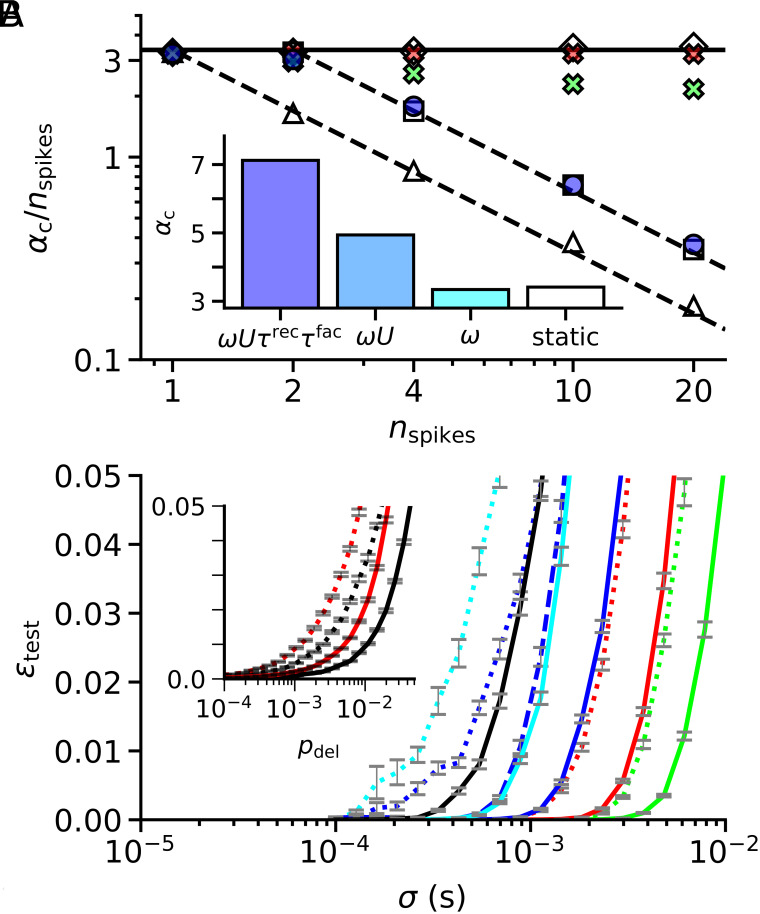
Storage capacity. (*A*) Normalized storage capacity $\alpha_{c}/n_{\mathrm{spikes}}$ for a neuron with N=100 afferents as a function of the number of input spikes per afferent $n_{\mathrm{spikes}}$ for different synapse models: Static synapse (1-weight case, open triangles), dynamic Tsodyks–Markram synapse (blue circles), and ordinal synapse with (green exes) and without (red exes) sign constraint. For reference, open diamonds show the normalized capacity for the $n_{\mathrm{spikes}}$-weight case and open squares for the 2-weight case (see text for details). The solid horizontal line depicts the average normalized capacity of the $n_{\mathrm{spikes}}$-weight reference case (open diamonds). The two dashed lines depict decays that are inversely proportional to $n_{\mathrm{spikes}}$ starting at the solid horizontal lines at $n_{\mathrm{spikes}}=1$ and $n_{\mathrm{spikes}}=2$, respectively. Fitting of the relation $\alpha_{c}/n_{\mathrm{spikes}}=\alpha_{1}n_{\mathrm{spikes}}^{\lambda }$ to the shown data points resulted in the following values: ($\alpha_{1}=3.26$, λ=0.02) $n_{\mathrm{spikes}}$-weight (open diamonds), ($\alpha_{1}=6.39$, λ=−0.96) 2-weight (open squares), ($\alpha_{1}=3.28$, λ=−0.97) 1-weight (open triangles), ($\alpha_{1}=3.26$, λ=0) ordinal synapses without sign constraint (red exes), ($\alpha_{1}=3.23$, λ=−0.14) ordinal synapse with sign constraint (green exes), ($\alpha_{1}=6.89$, λ=−0.98) Tsodyks–Markram synapse (blue circles). Because of its slow saturation, the fit of Tsodyks–Markram synapse was based only on data points with $n_{\mathrm{spikes}}\geq 4$. The *Inset* shows the storage capacity of the Tsodyks–Markram model for $n_{\mathrm{spikes}}=4$ in comparison to the static synapse (1-weight case, open bar, “static”), when trained fully (dark blue, “$\omega U\tau^{\mathrm{rec}}\tau^{\mathrm{fac}}$”) or partially, only ω and U (mid blue, “ωU”), or only ω (light blue, “ω”). (*B*) Robustness to noise. Mean generalization error $\varepsilon_{\mathrm{test}}$ (*Materials and Methods*) of neurons with dynamic (Tsodyks–Markram model, solid lines) and static synapses (1-weight case, dotted lines) at different learning loads α (α=0.5, green; α=1, red; α=2, dark blue; α=4, black) as a function of temporal input spike jitter σ. The light blue lines depict a load of α=2 but with overlapping input patterns (*Materials and Methods*) and the dashed dark blue line in between the dotted and the solid dark blue lines shows the robustness of a neuron whose dynamic synapses were only partially trained (ω and U). Gray error bars depict ± one SE of the mean. The *Inset* shows the mean generalization error (line styles as in the main Figure) for a load of α=2 as a function of spike deletion probability $p_{\mathrm{del}}$ (x-axis) with (red) and without (black) background firing (*Materials and Methods*).

We used the tempotron learning rule (3) to measure the storage capacity of a leaky integrate-and-fire neuron in the above scenarios (*Critical Capacity*). Reproducing previous work (3, 5), we found that the storage capacity in the one-spike-per-afferent scenario remained constant when we increased the number of afferents. Note, that for comparison with the dynamic synapses, we interpret this constant capacity as the normalized capacity, $\alpha_{c}/n_{\mathrm{spikes}}$ (cf. Eq. **1**), of a neuron in a multiple-spikes-per-afferent scenario that has a fixed number of afferents N and a variable number of input spikes $n_{\mathrm{spikes}}$ (fitted slope: 0.02, Fig. 2*A*, open diamonds). In the multiple-spikes-per-afferent scenario with static synapses, where each of the $n_{\mathrm{spikes}}$ spikes of the N afferents was weighted with the same static synaptic efficacy, we found that the number of patterns that the neuron could store did not increase with $n_{\mathrm{spikes}}$ but rather remained constant, so that the normalized capacity $\alpha_{c}/n_{\mathrm{spikes}}$ decreased inversely proportional to $n_{\mathrm{spikes}}$ (fitted slope: −0.97, Fig. 2*A*, open triangles). The above multiple- and single-spikes-per-afferent scenarios represent two limiting cases in which N groups of $n_{\mathrm{spikes}}$ spikes were either all arriving on one single afferent (1-weight case) or distributed over $n_{\mathrm{spikes}}$ afferents ($n_{\mathrm{spikes}}$-weight case), respectively. We also tested the intermediate case where the $n_{\mathrm{spikes}}$ spikes of each group were distributed over two afferents (2-weight case), each of which fired $n_{\mathrm{spikes}}/2$ spikes. In this scenario the number of patterns that the neuron could store increased by a factor of two (6.39/3.26 = 1.96) relative to the 1-weight case (fitted slope: −0.96, Fig. 2*A*, open squares). While a neuron with static synapses is able to decode temporal information that is encoded in the relative timing of spikes across space (3), i.e. different afferents, its ability to do so when the information is encoded within spike sequences of individual afferents is limited. Our results expose an intriguing limit of static synaptic transmission: The capacity of a neuron with static synapses to classify spike latency patterns cannot be increased by reusing its afferents within a processing time interval through additional spikes.

### Scaling Capacity with the Number of Input Spikes.

We next turn to the ordinal synapse scenario: We generated latency patterns such that the neuron’s $N\times n_{\mathrm{spikes}}$ afferents formed N groups of $n_{\mathrm{spikes}}$ afferents whose spikes had a fixed rank order across all input patterns. By how much would this order constraint that reduced the variability between different latency patterns degrade the neuron’s storage capacity? Intriguingly, in the biologically relevant regime of these parameters, i.e. N on the order of hundreds or thousands and $n_{\mathrm{spikes}}$ on the order of one or ten (corresponding to firing rates on the order of one to tens of Hertz), the effect of the ordering constraint was negligible (fitted slope: 0.0, Fig. 2*A*, red exes). Hence, in contrast to static synapses, plastic short-term plasticity can in principle allow the storage capacity of neurons in the brain to scale with the number of input spikes. The idealized ordinal synapse introduced above is free to assign different signs to spikes arriving on the same afferent. For example, the efficacy of the first spike of a given afferent could be excitatory, but the second inhibitory. Although there have been reports of corelease of excitatory and inhibitory transmitters (e.g. glutamate and GABA) at specialized synaptic connections (e.g. ref. 52), such flexibility seems implausible for most synaptic connections in the brain. We therefore checked, if the ability to switch back and forth between excitation and inhibition was crucial for the capacity of the ordinal synapse to scale with the number of input spikes. Specifically, we tested a sign constrained version of the ordinal synapse that was free to assign individual magnitudes to successive spikes but had to choose a single sign for all of them (*Sign-Constrained Ordinal Synapse*). Not surprisingly, the overall capacity of the sign constrained ordinal synapse was somewhat reduced. However, we found that the important increase in storage capacity with the number of input spikes per afferent was only slightly sublinear (fitted slope: −0.14, Fig. 2*A*, green exes).

### Phenomenological Short-Term Plasticity.

Short-term plasticity at biological synapses results from the dynamics of the molecular transmission machinery whose processes operate on time scales that range from fast fusion of docked vesicles in the submillisecond regime to the much slower retraction, refilling and tethering of vesicles or the buffering of presynaptic calcium on times scales of up to seconds (12, 14, 15, 53). The ability of a biological synapse to match the capabilities of an ordinal synapse and to adjust the efficacy of each spike in a sequence independently, depends on the richness of its dynamics and its ability to tune these dynamics through processes of long-term plasticity. While we expect the performance of a biologically plausible model of short-term plasticity to lie within the bounds set by the ordinal and the static synapses, we wanted to test to what degree already a simple model of short-term plasticity can realize the theoretically possible enhancement of storage capacity. We derived the tempotron learning rule for a leaky integrate-and-fire neuron with short-term plasticity as specified by the well-established Tsodyks–Markram model (30, 39, 42, 54, 55), which represents a good compromise between mathematical tractability and biological plausibility.

At its core, this phenomenological model consists of two dynamical state variables that subserve synaptic facilitation and depression through a facilitating utilization variable u, mimicking the accumulation of presynaptic calcium, and the concomitant depletion of available synaptic resources x, e.g. the size of the readily releasable pool. In between presynaptic spikes, both variables relax exponentially to their baseline values: u with facilitation time constant $\tau_{\mathrm{fac}}$ and x with recovery time constant $\tau_{\mathrm{rec}}$. In addition to the two time constants, the utilization parameter U and an overall postsynaptic scale ω make up a set of four parameters that characterizes each individual synapse (*Dynamic Synapse*).

Interestingly, we found that this simple model of short-term plasticity saturated at roughly the same performance as a model that had two independent static synapses associated with each afferent, i.e. the 2-weight case (Fig. 2*A*, blue circles, open squares). Note, however, that the dynamic synapse required more than two input spikes within its input pattern to reach this capacity (*SI Appendix*, Fig. S1, N=100). Unlike the ordinal synapse where the efficacies of successive spikes are independent, the phenomenological Tsodyks–Markram model of short-term plasticity has a limited dynamic range that results from complex interactions between the spikes within the afferent’s activity history. Nevertheless, for each number of input spikes per afferent the capacity was extensive, i.e. the number of input patterns that could be stored scaled linearly with the number of afferents (*SI Appendix*, Fig. S1). Hence, the increase in storage capacity due to the plastic short-term plasticity of the synapse was independent of the spatial scaling with the number of afferents N. Importantly, the enhanced storage capacity of the Tsodyks–Markram model required the cotraining of all synaptic parameters for a given batch of input patterns. While untrained short-term plasticity has been reported to increase a neuron’s temporal selectivity (50), its capacity returned to baseline when training was limited to the postsynaptic scale ω only. In this control, the initial values of ω and the other, untrained, synaptic parameters were taken from a solution obtained by training on an independent batch of patterns of the same statistics

(Fig. 2*A*, *Inset*). Partial training of other subsets of the synaptic parameters, e.g. training only ω and U, resulted in intermediate storage capacities (Fig. 2*A*, *Inset*).

### Robustness to Noise.

Basic machine learning intuition posits that for a given task (with fixed learning load) an increase in storage capacity goes hand in hand with larger decision margins, i.e. the existence of solutions that allow for greater robustness to noise. In the brain, one prominent source of noise is spike time jitter. Intuitively, temporal noise seems at conflict with the present mechanism that increases storage capacity by tuning the short-term plasticity of each synapse to the timing of its spikes. We therefore asked whether the observed increase in storage capacity can nevertheless support the enhancement of robustness with respect to temporal spike jitter. We found this to be the case: The increased storage capacity of neurons with Tsodyks–Markram synapses substantially enhanced their robustness to spike time jitter within their input activity (Fig. 2*B*). Specifically, for input patterns with four spikes per afferent, neurons with dynamic synapses could operate with similar error rates at nearly twice the learning loads as neurons with static synapses. This effect persisted for input patterns with elevated overlap and also when noise was generated by synaptic failures instead of temporal spike jitter (Fig. 2*B*, *Inset*; *Robustness to Noise*). Interestingly, the error curve corresponding to neurons with partially trained dynamical synapses (ω and U) lay roughly halfway between the curves of the static and the fully trained models, mirroring the relations between their storage capacities (Fig. 2). These findings underline that the ability of a neuron to tune the short-term plasticity of its synapses cannot only subserve a higher storage capacity but can also be traded for greater robustness with respect to noise, even in the time domain.

### Decoding Spatiotemporal Correlations.

The improved performance in discriminating spike latency patterns in the storage capacity task reflects the general ability of neurons with plastic short-term plasticity to decode information embedded in unstructured sequences of spike times of individual afferents. However, much of neural information processing in the brain involves detecting and responding to structured relationships—across space and time—between features within an organism’s sensory environment. To explore whether, and if so how, plastic short-term plasticity also improves a neuron’s ability to perform such relational processing, we extended a previously established task in which neurons with static synapses learned to discriminate spatial patterns of spike synchrony (3).

**Fig. 3.**
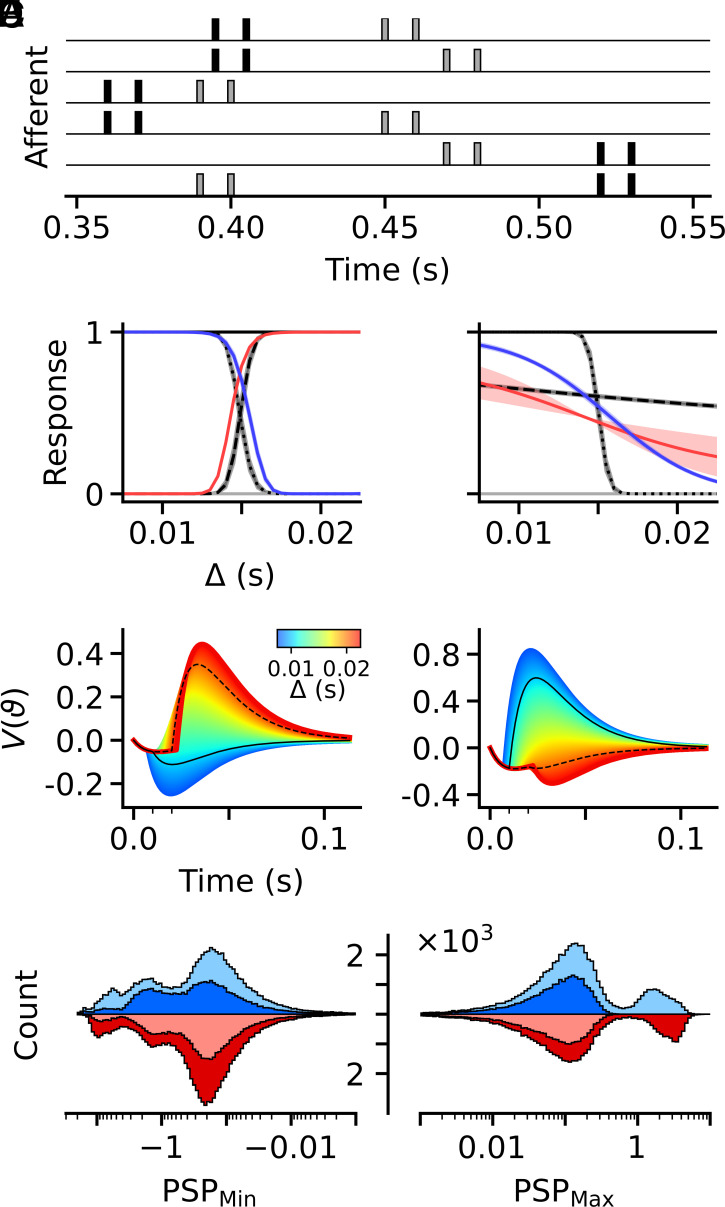
Decoding spatiotemporal correlations. (*A*) Schematic example of two input patterns (black and gray) with each afferent firing two spikes per pattern: the first at a random time and the second with a delay of Δ thereafter. Each pattern realizes one of two possible pairings of the input afferents that fire in synchrony: In one pairing (black), pairs are formed by afferents 1&2, 3&4, and 5&6; in the other (gray) by 1&4, 2&5, and 3&6. (*B* and *C*) Mean responses of neurons with dynamic (*B*) and static (*C*) synapses after learning for different values of the delay Δ (x-axis). Pairs of lines correspond to the two pairings in each of the four different tasks: pairing task—solid black (*Top*) and solid gray (*Bottom*); XOR task: red and blue. In the two delta tasks, short (dotted) and long (dashed), lines from the two pairings are indistinguishable. Lines depict averages over 100 independent simulations and shaded areas ±1sd. (*D* and *E*) Pair PSP traces of two example pairs of dynamic synapses for delays (color bar) ranging from Δ=7.5ms (blue) to Δ=22.5ms (red). Black lines depict pair PSPs for the two task delays (minor x-ticks) of 10ms (solid) and 20ms (dashed). (*F*) Stacked histograms of pair PSP minima (*Left*) and maxima (*Right*) of neurons with dynamic synapses that were trained to solve the XOR task. Colors blue (upward) and red (downward) represent the two pairings, and brightness levels dark and light represent the short and long delay conditions. In the XOR task, PSP distributions of target patterns are depicted by light blue and dark red histograms and PSP distributions of null patterns by dark blue and light red. Histograms are computed over synapses of 1,000 independently trained neurons, excluding maxima (45%) and minima (20%) with absolute magnitudes below $10^{-3}$.

All N afferents of a spike latency pattern were grouped into N/2 distinct pairs, each of which fired synchronous spikes at times t and t+Δ within a given pattern. For each pair, the time of the first spikes was drawn independently from a uniform distribution over the interval from 0 and 500 ms, and the delay Δ to the second spikes was the same across all pairs (Fig. 3*A*) in a pattern. Using two fixed pairings and two fixed delays, a short delay (Δ=10ms) and a long delay (Δ=20ms), we defined four binary classification tasks: The first, the pairing task, was spatial and required neurons to respond to one of the two pairings of the afferents but not to the other, irrespective of the delay. The second and third tasks were temporal: In the short-delay task, neurons had to respond to patterns with the short delay but not to the long one, irrespective of the pairing; in the long-delay task, the requirement was reversed. The fourth task combined both spatial and temporal contingencies: In this XOR-like task, neurons had to respond to the short delay for one pairing and to the long delay for the other, while remaining silent for the opposite combinations.

We found that neurons with plastic short-term plasticity were able to learn all four tasks with high accuracy, whereas neurons with static synapses failed to learn the long-delay and XOR tasks (Fig. 3 *B* and *C*; *Spatiotemporal Correlation Task*). Given the linear voltage integration of the neuron model, these failures are intuitive: For static synapses, the peak amplitudes (magnitudes) of the summed postsynaptic potentials (PSPs) generated by the two spikes of each afferent decrease monotonically with increasing Δ. The same holds for all pairwise PSPs, i.e. the combined postsynaptic responses of synchronously firing pairs of afferents. However, for neurons with dynamic synapses, the situation is more complex because the efficacy of the second spike from an individual afferent generally differs from that of the first. To illustrate this, consider a pair of afferents consisting of one excitatory and one inhibitory synapse of approximately equal strength, such that their responses to the first spike roughly cancel. If both synapses are depressing, but the excitatory one depresses more strongly and recovers more rapidly than the inhibitory one, then inhibition will dominate for small Δ, whereas excitation will dominate when Δ is large (Fig. 3*D*). Conversely, if the inhibitory synapse depresses more strongly but recovers more rapidly than the excitatory one, the resulting pair PSP will be excitatory for small values of Δ and inhibitory for large ones (Fig. 3*E*). To illustrate the mechanism underlying the solution of the XOR task, we computed histograms of the maxima and minima of pairwise PSPs across all four task conditions (Fig. 3*F*). Consistent with the neuron’s firing behavior after learning, these histograms showed distinct peaks corresponding to large PSP maxima in the two target conditions, which were absent in the two null conditions.

### Comparison to Physiological Short-Term Plasticity.

**Fig. 4.**
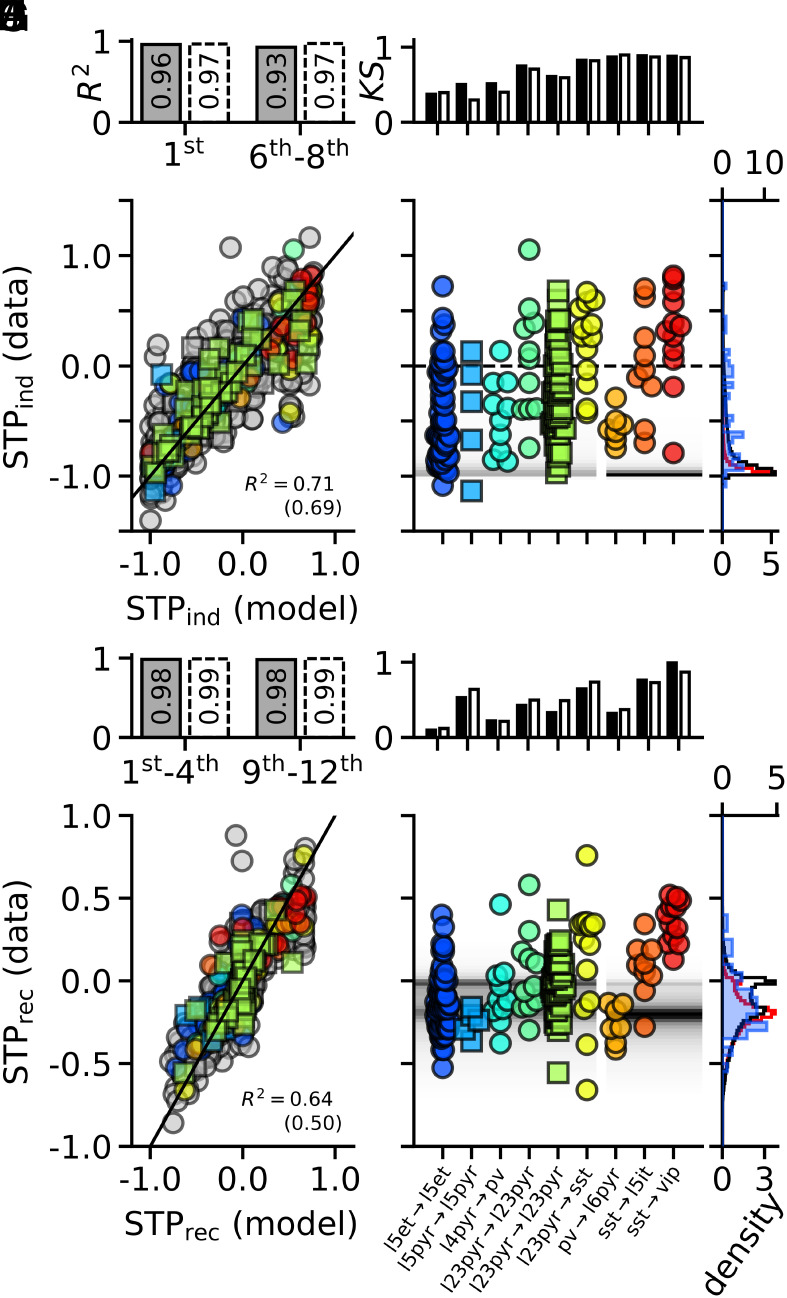
Comparison to electrophysiology. *Top* panels (*A*–*E*) refer to short-term plasticity induction; *Bottom* panels (*F*–*J*) refer to recovery. (*A*) $R^{2}$ values of the Tsodyks–Markram model for the first (1st, *Left*) and the late (6th to 8th, *Right*) average responses (*Materials and Methods*). Fill color and edge styles indicate data from mice (filled, solid) and humans (open, dashed). (*B*) Scatter plot of short-term plasticity induction of electrophysiological connections [y-axis, ${\mathrm{STP}}_{\mathrm{ind}}$ (data)] versus their fitted Tsodyks–Markram counterparts [x-axis, ${\mathrm{STP}}_{\mathrm{ind}}$ (model)]. Circles represent connections from mice ($R^{2}$ value *Lower Right*), and squares connections from humans ($R^{2}$ value *Lower Right* in parentheses). The solid diagonal depicts the identity line and colors other than gray highlight groups of connections of the specific types shown in (*D*). (*C*) Kolmogorov–Smirnov test statistics (*Materials and Methods*) between the samples of short-term plasticity induction of the connection-types shown in (*D*) and the corresponding distribution of short-term plasticity induction of Tsodyks–Markram synapses trained in the classification task (*D*, background). Black bars correspond to electrophysiologically observed connections whereas open bars correspond to their fitted Tsodyks–Markram counter parts. (*D*) Short-term plasticity induction values (y-axis) of individual connections of specific types [x-axis, naming following (40)]. Plot symbols and colors as in (*B*). Gray levels in the background show the corresponding distribution of short-term plasticity induction of excitatory (*Left*) and inhibitory (*Right*) Tsodyks–Markram synapses (*E*). The dashed horizontal line at 0 separates short-term facilitation (*Above*) from short-term depression (*Below*). (*E*) Distribution of short-term plasticity induction of Tsodyks–Markram synapses trained in the classification task with $n_{\mathrm{spikes}}=4$ and load α=6.7 (excitatory connections: black, *Bottom* x-axis; inhibitory connections: red, *Top* x-axis). For comparison the blue histogram (*Bottom* x-axis) shows the distribution of short-term plasticity induction values of l5et→l5et connections (leftmost swarm in *D*). (*F*), as in (*A*) but for average initial responses (1st to 4th, *Left*) and average recovered responses (9th to 12th, *Right*). (*G*–*J*) as in (*B*–*E*), but for short-term plasticity recovery (${\mathrm{STP}}_{\mathrm{rec}}$) instead of induction (${\mathrm{STP}}_{\mathrm{ind}}$).

Already the simple phenomenological Tsodyks–Markram model gives rise to a remarkable enhancement of a circuit’s learning capacity and robustness to noise. To test the validity of the model, we fitted the short-term plasticity of recently reported electrophysiological recordings of synaptic connections in mouse and human neocortex (40, 51). Following ref. 40, we concentrated on two measures of short-term plasticity that were computed on the basis of 4 groups of postsynaptic responses within a sequence of 12 stimulation pulses, namely the first pulse (1st), pulses 1 through 4 (1st to 4th), pulses 6 through 8 (6th to 8th), and pulses 9 to 12 (9th to 12th). The two measures were, first, the induction of short-term plasticity (${\mathrm{STP}}_{\mathrm{ind}}$) through repetitive stimulation and, second, the recovery from short-term plasticity (${\mathrm{STP}}_{\mathrm{rec}}$) following a pause in stimulation (*Electrophysiological Data Analysis*, Eqs. **18** and **19**). While induction was measured by the change between the initial (1st) and late (6th to 8th) responses, recovery was measured by changes between the first four (1st to 4th) and the last four (9th to 12th) responses that were delivered after a stimulation pause of 250ms. For both measures, differences were normalized by the maximal response of a given connection over all 12 stimulation pulses (*Electrophysiological Data Analysis*). Average responses over each of the four groups were fitted by the Tsodyks–Markram model with high accuracy (Fig. 4 *A* and *F*). As a result, the Tsodyks–Markram synapses were able to reliably replicate the induction and recovery of the experimentally observed short-term plasticity (Fig. 4 *B* and *G*).

We next simulated the experimental protocol to measure the distributions of short-term plasticity induction and recovery within populations of Tsodyks–Markram synapses that were trained on the storage capacity task studied above (Fig. 4 *E* and *J*). We separated synapses into excitatory and inhibitory populations based on the sign of their efficacy w. Near the critical capacity, the distributions of short-term plasticity induction for both excitatory and inhibitory connections were concentrated close to the lower bound of −1, resembling a dominance of strong short-term depression (Fig. 4*E*). This trend was particularly pronounced for inhibitory connections (red line, *Top* x-axis). Meanwhile, the distribution of recovery from short-term plasticity showed two peaks, one near 0 for synapses that were fully recovered and a second one at approximately −0.2 stemming from partially recovered synapses whose learned recovery time constants exceeded the 250ms recovery time interval of the experimental protocol (Fig. 4*J*). While the peak at −0.2 was present in excitatory and inhibitory connections, the peak at 0 was more prominent in the excitatory population. Intriguingly, when we compared the distributions of synaptic behaviors between specific experimentally observed connection types and Tsodyks–Markram synapses with matching synaptic polarity, we found that for both measures, excitatory cortical layer 5 connections in mice showed the closest resemblance (Fig. 4*C*, *D*, *H*, and *I*). While the distributions of recovery showed remarkable quantitative agreement (Fig. 4*J*), the agreement of induction was only qualitative, in the sense that a large majority of the measured connections were depressing (84%). However, rather than being concentrated at the limiting value of −1 (as were the model synapses), the levels of depression of measured connections were scattered almost uniformly between 0 and −1. This discrepancy could result from cortical connections not operating as close to critical capacity as the ones in our simulations. Nevertheless, these comparisons show that the improved capacity of neurons with learned synaptic dynamics is subserved by biologically plausible distributions of short-term plasticity induction and recovery.

## Discussion

Our results demonstrate a perplexing limitation of static synaptic transmission: Increasing the number of spikes within spike latency patterns does not increase a circuit’s storage capacity. This limitation can be overcome, if a circuit is endowed with plastic short-term plasticity. Specifically, we show that plastic short-term plasticity enables neurons to process sequences of spikes of a given afferent as if they were spatial patterns across as many afferents as there are spikes in the sequence. Already neurons with the simple phenomenological Tsodyks–Markram model of short-term plasticity can utilize this temporal degree of freedom to double their storage capacity or substantially increase their robustness to noise. These findings are not limited to the Tsodyks–Markram model, as is underlined by the higher capacity of the two idealized ordinal synapse models. We expect that more complex models of short-term plasticity than the Tsodyks–Markram model, with richer dynamics, for instance through multiple time constants for facilitation (44) or depression (56), will further improve storage capacity toward the capacity of ordinal synapses.

Realization of these capabilities requires synapses to individually tune their short-term plasticity. While interactions between short- and long-term plasticity are well established (29, 42–44, 57–59), systematic learning rules that govern such changes have yet to be studied and uncovered. Besides the important challenge to precisely control supervisory signaling in electrophysiological preparations, the development of adequate plasticity induction protocols is critical. Specifically, our model highlights the importance of controlling the dynamic state of the synapse at the time of plasticity induction and the time course of the postsynaptic membrane potential, e.g. through pre- and or postsynaptic burst firing. Typical pairing protocols for the induction of plasticity have used pre- and postsynaptic current injections over hundreds of milliseconds without controlling the specific dynamic state of the connection or the time course of the postsynaptic membrane potential (43, 57, 59). Other induction protocols have used repetitions of isolated pre- and postsynaptic spike pairs (58) without engaging synaptic dynamics. These experiments have yielded diverse results following the induction of long-term potentiation, ranging from short-term plasticity becoming more depressing (43, 57, 59), remaining unchanged (57, 59) to becoming less depressing (59). It has been suggested that this diversity could result from different combinations of various forms of pre- and postsynaptic long-term potentiation and depression (58, 59) that are expressed at a given connection. In addition to specifically tailored experimental induction protocols, quantitative comparisons of changes in short-term plasticity between experiments and computational models, also require the constraining of the exact parameterization of the voltage gradients that underlie the theoretical learning rules (60). For example, while the original Tsodyks–Markram model can be consistent with both positive and negative correlations between changes in the postsynaptic scale ω and the utilization U (*SI Appendix*, Fig. S2, *Middle* row), the gradient of the refactored model derived in the present study (*SI Appendix*, Fig. S3, *Middle* row) is only consistent with forms of plasticity where this correlation is negative. Both versions of the model predict a negative correlation between changes in ω and the recovery time constant $\tau^{\mathrm{rec}}$, in contrast to recent experimental findings that describe the emergence of an additional slow time scale following the induction of long-term potentiation at layer 5 pyramidal neurons (56) that lies outside the scope of the present model. Note, however, that we have used the simple phenomenological Tsodyks–Markram model mainly to uncover the basic ability of biologically plausible models of short-term plasticity to subserve the enhancement of storage capacity in neural circuits. In future work, it will be important to extend our theory toward recent mechanistic models of short-term plasticity that incorporate reversible multistep tethering, priming, and fusion processes (41, 61) and involve a much larger number of interaction sites between short- and long-term plasticity (53).

Nevertheless, we have shown that, despite its simplicity, the Tsodyks–Markram model is capable of reproducing the physiologically observed behavior of cortical connections with high accuracy. Moreover, a connection type specific comparison revealed an intriguing qualitative match between the synaptic behaviors of layer 5 excitatory connections and Tsodyks–Markram synapses that were trained to classify multiple-spikes-per-afferent spike latency patterns.

Our work has uncovered two important computational consequences of plastic short-term plasticity: First, it enables neurons to decode multineuronal spike correlations that extend over space and time, and second, it can subserve a computational paradigm for neural networks in which the effective amount of wiring of a neuronal architecture is not static but modulated on demand through the level of activity that an input layer commits to a given task.

## Materials and Methods

### Neuron Model.

Our study is based on the standard current-based leaky integrate-and-fire model (e.g., ref. 3). In this model, the neuron’s membrane potential results from the integration of exponentially decaying input currents that are elicited by the spiking activity of the neuron’s N afferents. Specifically, the membrane potential at time t

[2] $$\begin{array}{c} V\left(t\right)=\sum_{i=1}^{N}\sum_{t_{i}^{j}<t}W_{i}\left(\left\{t_{i}^{1},t_{i}^{2},\ldots ,t_{i}^{j}\right\}\right)K\left(t-t_{i}^{j}\right)+V_{\mathrm{rest}} \end{array}$$

is given by summing over all incoming spikes (indexed by j) of each afferent i=1,⋯,N with arrival times $t_{i}^{j}<t$. Each of these spikes contributes a unitary postsynaptic potential $K\left(t-t_{i}^{j}\right)=V_{0}\cdot [\exp\left(-\frac{t-t_{i}^{j}}{\tau_{m}}\right)-\exp\left(-\frac{t-t_{i}^{j}}{\tau_{s}}\right)]$ that is weighted by the afferent’s instantaneous synaptic efficacy $W_{i}\left(\left\{t_{i}^{1},t_{i}^{2},\ldots ,t_{i}^{j}\right\}\right)$. In contrast to previous formulations of this model where synaptic efficacies were characterized by scalar weights, the present model allows each synaptic efficacy to depend on the afferent’s activity history. The postsynaptic potentials $K(t-t_{i}^{j})$ are normalized to unit amplitude by setting

[3] $$\begin{array}{c} V_{0}=\frac{\tau_{s}}{\tau_{m}-\tau_{s}}{\left(\frac{\tau_{m}}{\tau_{s}}\right)}^{\frac{\tau_{m}}{\tau_{m}-\tau_{s}}}, \end{array}$$

where $\tau_{m}$ and $\tau_{s}$ denote the membrane and synaptic time constants. The postsynaptic potentials are causal, i.e. $K(t-t_{i}^{j})$ vanishes for $t<t_{i}^{j}$. Whenever the membrane potential V crosses the spike threshold at ϑ=1, the neuron elicits an output spike, and the voltage undergoes a smooth reset to $V_{\mathrm{reset}}=V_{\mathrm{rest}}=0$ by shunting all incoming spikes that arrive after the output spike (3).

### Integration Time Scales.

The membrane and synaptic time constants $\tau_{m}$ and $\tau_{s}$ determine the shape of the unitary postsynaptic potentials $K(t-t_{i}^{j})$. Since the overall effect of their ratio $\tau_{m}/\tau_{s}$ on the capacity of the model is weak in the biologically common range between 1 and 4, we kept $\tau_{m}=2\tau_{s}$ throughout this work. In contrast, the capacity of the model is sensitive to the characteristic temporal scale $\tau \equiv \sqrt{\tau_{m}\tau_{s}}$ that governs the effective temporal extent of the postsynaptic potentials and has to be matched to the density of input spikes for optimal performance (see also ref. 3). Specifically, we used $\tau =5.67T/(Nn_{\mathrm{spikes}})$ where T is the duration of input patterns and $n_{\mathrm{spikes}}$ denotes the total number of spikes per afferent in a given task.

### Storage Capacity Task.

This binary classification task requires a neuron to respond differently to two classes, target and null, that belong to a fixed batch of p input patterns. Specifically, the neuron is required to fire at least one action potential in response to a target pattern but it must remain silent in response to a null pattern. Implementing a storage capacity task where no generalization is achievable, target and null patterns are drawn iid from the same distribution of spike times and designated randomly, with equal probability, as target or null. Each of the N afferents of an input pattern fires $n_{\mathrm{spikes}}$ spikes at times $\left\{t_{i}^{j}\right\}$ (i=1,⋯,N; $j=1,\cdots ,n_{\mathrm{spikes}}$) that are drawn independently from a uniform distribution over the pattern duration T=500ms. Note that, because each afferent fires exactly $n_{\mathrm{spikes}}$ spikes in each pattern, these spike latency patterns cannot be discriminated on the basis of spike counts. Instead, successful classification requires the readout of spike timing. In Fig. 2*B* we also tested the neurons’ robustness to noise for patterns with overlap. Each batch of p patterns with overlap was generated by jittering the spike times of p copies of one spike latency pattern with additive Gaussian noise with a SD of 10 ms. For implementations of the independent and ordinal synapses, multiple-spikes-per-afferent patterns are transformed into single-spike-per-afferent patterns over $N\times n_{\mathrm{spikes}}$ afferents. While the spike times of these $N\times n_{\mathrm{spikes}}$ afferents are independent in the independent synapse case (Fig. 1*B*) their rank order is constrained for ordinal synapses. Specifically, in the ordinal synapse case afferents are assigned to N groups of $n_{\mathrm{spikes}}$ afferents within which the spike times are sorted, such that their rank order remains fixed within each group across all input patterns (Fig. 1*D*). Our estimates of the storage capacity of neurons with different synapse models are based on their convergence times on this task. Specifically, for a given learning load α we define convergence time as the number of batch iterations that is required until the neuron classifies all p=αN patterns correctly.

### Spatiotemporal Correlation Task.

Training in all four tasks proceeded by randomly choosing, with equal probabilities, one of the two parings and one of the two delays for each new training pattern and assigning the pattern to the target or null class in accordance with the task contingencies. To improve generalization, Gaussian noise with zero mean and a SD of 1 ms was added to the value of the delay for each training pattern. Training extended over one hundred thousand learning cycles, each containing ten thousand training patterns. Response curves (Fig. 3 *B* and *C*) were acquired and averaged over measurements in between each of the last one thousand learning cycles using ten thousand test patterns per point. Pair PSPs in Fig. 3 *D* and *E* were computed for two exemplary synapse pairs taken from opposing pairings after training a neuron on the XOR task. The pair in Fig. 3*D* has parameters: ω=5.97, $\omega^{\prime }=-6.02$; U=0.86, $U^{\prime }=0.78$; $\tau_{\mathrm{rec}}=0.050$, $\tau_{\mathrm{rec}}^{\prime }=0.112$; $\tau_{\mathrm{fac}}=0.084$, $\tau_{\mathrm{fac}}^{\prime }=0.063$; and the one in Fig. 3*E* has ω=5.44, $\omega^{\prime }=-5.62$; U=0.68, $U^{\prime }=0.93$; $\tau_{\mathrm{rec}}=0.113$, $\tau_{\mathrm{rec}}^{\prime }=0.034$; $\tau_{\mathrm{fac}}=0.048$, $\tau_{\mathrm{fac}}^{\prime }=0.090$.

### Robustness to Noise.

We tested the robustness of the neurons’ classification performance through a modified version of the above classification task. In this version, the input patterns of a given batch were never presented to the neuron directly but served as templates that were corrupted with independent noise before each pattern presentation. We used three different types of noise: First, temporal noise implemented by perturbing all spike times by additive Gaussian noise with zero mean and SD σ. Second, synaptic failures implemented by deleting each spike with probability $p_{\mathrm{del}}$. And last, synaptic failures with background firing, implemented by deleting each spike with probability $p_{\mathrm{del}}$ and adding Poissonian spikes with rate $p_{\mathrm{del}}\times n_{\mathrm{spikes}}$, such that, on average, the patterns’ spike counts remained unchanged. For each noise strength, learning proceeded for one million batch cycles during which learning curves (*SI Appendix*, Figs. S4 and S5) were acquired. In addition to the training errors over each batch cycle, we also measured the neuron’s average generalization error every fifty thousand cycles. Specifically, each of these measurements averaged the generalization error over one thousand consecutive batch learning cycles. After each learning cycle, the error was evaluated by cyclically sampling ten thousand patterns from the batch without learning. Values shown in Fig. 2*B* represent averages of the final average generalization errors over 50 independent realizations of each task.

### Tempotron Learning.

Tempotron learning (3) posits that neurons are trained by minimizing the single pattern cost function $E=\mp V(t_{\max})$ on each error trial where the minus and plus signs refer to target and null patterns, respectively. Here, $t_{\max}$ denotes the time of the maximum postsynaptic membrane potential. Following an error, all synaptic parameters that govern the instantaneous efficacies $W_{i}\left(\left\{t_{i}^{1},t_{i}^{2},\ldots ,t_{i}^{j}\right\}\right)$ are modified along the gradient of E. For example, for a synaptic parameter $\omega_{i}$, updates would be given by

[4] $$\begin{array}{c} \Delta \omega_{i}=\pm \eta_{\omega }\frac{dV(t_{\max})}{d\omega_{i}}, \end{array}$$

where $\eta_{\omega }$ is a parameter specific learning rate and the plus and minus signs correspond to updates that follow errors on target and null patterns, respectively.

Following ref. 3, we employ a momentum heuristic to accelerate convergence in all learning tasks. Specifically, synaptic updates are given by the combination of steps along the gradient (Eq. **4**) and the previous update

[5] $$\begin{array}{c} \Delta \omega_{i}^{\mathrm{current}}=\Delta \omega_{i}+\mu_{\omega }\Delta \omega_{i}^{\mathrm{previous}}. \end{array}$$

Here, the momentum parameter $\mu_{\omega }$ determines the fraction of the previous update that is added to the gradient contribution. As a result, learning of each synaptic parameter is controlled by two hyperparameters, i.e. the learning rate η and the momentum parameter μ (*SI Appendix*, *Optimization of Learning Parameters*, *Implementation Details*, and *Bayesian Optimization*).

### Static Synapse.

In most previous studies of supervised learning in spiking neural networks (3–11) synaptic efficacies were assumed to be independent of an afferent’s recent activity history, i.e. did not express any short-term synaptic plasticity. In the present framework, this class of networks is realized by setting

[6] $$\begin{array}{c} W_{i}\left(\left\{t_{i}^{1},t_{i}^{2},\ldots ,t_{i}^{j}\right\}\right)=\omega_{i}, \end{array}$$

such that the instantaneous synaptic efficacies reduce to scalars and the standard model and tempotron learning rule are recovered (3). Note that in this original model, the momentum heuristic is only invoked if the gradient contribution to $\Delta \omega_{i}$ (cf. Eq. **4**) is nonzero; otherwise, $\omega_{i}$ and its previous update $\Delta \omega_{i}^{\mathrm{previous}}$ (Eq. **5**) remain unchanged.

### Ordinal Synapse.

Contrasting the above limit of static synaptic transmission the ordinal synapse represents the opposite extreme in which each of the successive spikes that arrive at an afferent is weighted with an independent synaptic efficacy that depends only on the rank order of the spike within the spike sequence, i.e.

[7] $$\begin{array}{c} W_{i}\left(\left\{t_{i}^{1},t_{i}^{2},\ldots ,t_{i}^{j}\right\}\right)=\omega_{i}^{j}. \end{array}$$

As explained in the results, we implement the ordinal synapse by converting multiple-spikes-per-afferent patterns to single-spike-per-afferent patterns of higher dimension whose arrival times obey a temporal order constraint. The resulting model is trained with the learning rule for static synapses.

### Sign-Constrained Ordinal Synapse.

In addition to the above ordinal synapse (Eq. **7**) that is free to switch between excitatory and inhibitory efficacies for individual spikes of a single afferent, we also study a sign-constrained ordinal synapse, where all efficacies of a given afferent are constrained to have the same sign. Specifically, for patterns with $n_{\mathrm{spikes}}$ spikes per afferent the instantaneous efficacies

[8] $$\begin{array}{c} W_{i}\left(\left\{t_{i}^{1},t_{i}^{2},\ldots ,t_{i}^{j}\right\}\right)=\omega_{i}\;\frac{z_{i}^{j}}{\left|\right|\vec{z_{i}}{\left|\right|}_{1}} \end{array}$$

result from the product of an afferent specific scale, $\omega_{i}$, that also carries the afferent’s sign and a spike-order-dependent nonnegative magnitude $z_{i}^{j}\geq 0$. The L1 norm $\left|\right|\vec{z_{i}}{\left|\right|}_{1}=\sum_{i=1}^{n_{\mathrm{spikes}}}\left|z_{i}^{j}\right|$ normalizes the spike-order-dependent magnitudes $\vec{z_{i}}=\left(z_{i}^{1},z_{i}^{2},\ldots ,z_{i}^{n_{\mathrm{spikes}}}\right)$.

Implementing tempotron learning for the sign-constrained ordinal synapse, we obtain

$$\begin{array}{c} \frac{dV(t_{\max})}{d\omega_{i}}=\frac{1}{\left|\right|\vec{z_{i}}{\left|\right|}_{1}}\sum_{t_{i}^{j}<t_{\max}}z_{i}^{j}K\left(t_{\max}-t_{i}^{j}\right) \end{array}$$

and

$$\begin{array}{c} \frac{dV(t_{\max})}{dz_{i}^{j}}=\frac{\omega_{i}}{\left|\right|\vec{z_{i}}{\left|\right|}_{1}}\left[K\left(t_{\max}-t_{i}^{j}\right)-\frac{dV(t_{\max})}{d\omega_{i}}\right] \end{array}$$

as gradient of V with respect to ω and z at time $t_{\max}$. In this model, we employ the same momentum heuristic, as defined for the static synapse, independently for each $\omega_{i}$ and $z_{i}^{j}$.

### Dynamic Synapse.

The static and ordinal synapses correspond to two extreme cases of synaptic dynamics where either no dependence on previous spikes is present, the static synapse, or the efficacy of each spike can be chosen independently, the ordinal synapse. In addition to these limiting cases, we study the well-validated phenomenological Tsodyks–Markram model of short-term plasticity (30, 42) as an intermediate model that mimics the biomolecular dynamics of synaptic transmission in the central nervous system. In this model, the instantaneous synaptic efficacy is given by

[9] $$\begin{array}{c} W_{i}\left(\left\{t_{i}^{1},t_{i}^{2},\ldots ,t_{i}^{j}\right\}\right)=\omega_{i}u_{i}^{j}x_{i}^{j}, \end{array}$$

where $\omega_{i}$ denotes an overall static synaptic scale of the ith afferent and the product $u_{i}^{j}x_{i}^{j}$ represents the dynamic synaptic resources that are used by the jth spike of the ith afferent to generate the corresponding synaptic current. Specifically, the depressing variable $x_{i}^{j}$ mimics the amount of synaptic resources available to the jth incoming spike and the facilitating utilization variable $u_{i}^{j}$ determines the fraction of these resources that are released for each spike (25). The instantaneous synaptic efficacy is modulated by the time evolution of these two factors that depend on the number and timing of previous spikes. For the first spike of each afferent in an input pattern, both factors begin at unity, their baseline values,

[10] $$\begin{array}{c} u_{i}^{1}=1,\;x_{i}^{1}=1. \end{array}$$

Note that this formulation deviates slightly from the original model (25, 30, 42) (*SI Appendix*, *Original Tsodyks–Markram Model*) where the baseline value of the facilitating utilization variable $u_{i}^{j}$ corresponds to the utilization parameter is $U_{i}$ and carries an additional scale for the values of $u_{i}^{j}$ (separate from $\omega_{i}$). To enforce correspondence between the static and dynamic synapse models in the present work (for instance when $n_{\mathrm{spikes}}=1$, i.e. each afferent fires only one spike), we absorbed this redundant scale into $\omega_{i}$. This refactorization of the original model also improves the present gradient-based learning scheme by reducing interferences between updates to $\omega_{i}$ and $U_{i}$. Mimicking synaptic facilitation, e.g. through the accumulation of presynaptic calcium (25), each input spike following the first one, j>1, increases the utilization variable $u_{i}^{j}$ as

[11] $$\begin{array}{c} u_{i}^{j}=u_{i}^{j-1}\left(1-U_{i}\right)\exp(\frac{-\Delta t_{i}^{j}}{\tau_{i}^{\mathrm{fac}}})+1, \end{array}$$

where

$$\begin{array}{c} \Delta t_{i}^{j}=t_{i}^{j}-t_{i}^{j-1} \end{array}$$

denotes the time difference between the jth and the (j−1)th spikes and the facilitation time constant $\tau_{\mathrm{fac}}$ governs the return to baseline in the absence of input spikes. In turn, each release event depletes the available synaptic resources, such that for j>1, the depressing resource variable $x_{i}^{j}$ is given by

[12] $$\begin{array}{c} x_{i}^{j}=x_{i}^{j-1}\left(1-U_{i}u_{i}^{j-1}\right)\exp(\frac{-\Delta t_{i}^{j}}{\tau_{i}^{\mathrm{rec}}})+1-\exp(\frac{-\Delta t_{i}^{j}}{\tau_{i}^{\mathrm{rec}}}), \end{array}$$

where, in between spikes, $x_{i}^{j}$ returns to baseline with recovery time constant $\tau_{\mathrm{rec}}$.

To implement tempotron learning for the dynamic synapse we calculated the gradients of V with respect to its parameters ω, U, $\tau^{\mathrm{rec}}$ and $\tau^{\mathrm{fac}}$ at time $t_{\max}$, yielding the following derivatives:

[13] $$\begin{array}{cc} \frac{dV(t_{\max})}{d\omega_{i}} & =\sum_{t_{i}^{j}<t_{\max}}u_{i}^{j}x_{i}^{j}K\left(t_{\max}-t_{i}^{j}\right), \end{array}$$

[14] $$\begin{array}{cc} \frac{dV(t_{\max})}{dU_{i}} & =\omega_{i}\sum_{t_{i}^{j}<t_{\max}}K\left(t_{\max}-t_{i}^{j}\right)(u_{i}^{j}\frac{dx_{i}^{j}}{dU_{i}}+x_{i}^{j}\frac{du_{i}^{j}}{dU_{i}}) \end{array}$$

$$\begin{array}{c} \frac{du_{i}^{1}}{dU_{i}}=0 \end{array}$$

$$\begin{array}{c} \frac{du_{i}^{j}}{dU_{i}}=\;\exp(\frac{-\Delta t_{i}^{j}}{\tau_{i}^{\mathrm{fac}}})[\left(1-U_{i}\right)\frac{du_{i}^{j-1}}{dU_{i}}-u_{i}^{j-1}] \end{array}$$

for j≥2,

$$\begin{array}{c} \frac{dx_{i}^{1}}{dU_{i}}=0 \end{array}$$

$$\begin{array}{c} \frac{dx_{i}^{j}}{dU_{i}}=\;\exp(\frac{-\Delta t_{i}^{j}}{\tau_{i}^{\mathrm{rec}}})[\left(1-U_{i}u_{i}^{j-1}\right)\frac{dx_{i}^{j-1}}{dU_{i}}-x_{i}^{j-1}\left(u_{i}^{j-1}+U_{i}\frac{du_{i}^{j-1}}{dU_{i}}\right)] \end{array}$$

for j≥2,

[15] $$\begin{array}{c} \frac{dV(t_{\max})}{d\tau_{i}^{\mathrm{rec}}}=\omega_{i}\sum_{t_{i}^{j}<t_{\max}}u_{i}^{j}K\left(t_{\max}-t_{i}^{j}\right)\frac{dx_{i}^{j}}{d\tau_{i}^{\mathrm{rec}}} \end{array}$$

$$\begin{array}{cc} & \frac{dx_{i}^{1}}{d\tau_{i}^{\mathrm{rec}}}=0\\ \frac{dx_{i}^{j}}{d\tau_{i}^{\mathrm{rec}}} & =\;\exp(\frac{-\Delta t_{i}^{j}}{\tau_{i}^{\mathrm{rec}}})[\left(1-U_{i}u_{i}^{j-1}\right)(\frac{dx_{i}^{j-1}}{d\tau_{i}^{\mathrm{rec}}}+x_{i}^{j-1}\frac{\Delta t_{i}^{j}}{{\left(\tau_{i}^{\mathrm{rec}}\right)}^{2}})\\ & -\frac{\Delta t_{i}^{j}}{{\left(\tau_{i}^{\mathrm{rec}}\right)}^{2}}] \end{array}$$

for j≥2,

[16] $$\begin{array}{c} \frac{dV(t_{\max})}{d\tau_{i}^{\mathrm{fac}}}=\omega_{i}\sum_{t_{i}^{j}<t_{\max}}K\left(t_{\max}-t_{i}^{j}\right)(u_{i}^{j}\frac{dx_{i}^{j}}{d\tau_{i}^{\mathrm{fac}}}+x_{i}^{j}\frac{du_{i}^{j}}{d\tau_{i}^{\mathrm{fac}}}) \end{array}$$

$$\begin{array}{c} \frac{du_{i}^{1}}{d\tau_{i}^{\mathrm{fac}}}=0 \end{array}$$

$$\begin{array}{c} \frac{du_{i}^{j}}{d\tau_{i}^{\mathrm{fac}}}=\left(1-U_{i}\right)\exp(\frac{-\Delta t_{i}^{j}}{\tau_{i}^{\mathrm{fac}}})[\frac{du_{i}^{j-1}}{d\tau_{i}^{\mathrm{fac}}}+u_{i}^{j-1}\frac{\Delta t_{i}^{j}}{{\left(\tau_{i}^{\mathrm{fac}}\right)}^{2}}] \end{array}$$

for j≥2,

$$\begin{array}{c} \frac{dx_{i}^{1}}{d\tau_{i}^{\mathrm{fac}}}=0 \end{array}$$

$$\begin{array}{c} \frac{dx_{i}^{j}}{d\tau_{i}^{\mathrm{fac}}}=\;\exp(\frac{-\Delta t_{i}^{j}}{\tau_{i}^{\mathrm{rec}}})[\left(1-U_{i}u_{i}^{j-1}\right)\frac{dx_{i}^{j-1}}{d\tau_{i}^{\mathrm{fac}}}-U_{i}x_{i}^{j-1}\frac{du_{i}^{j-1}}{d\tau_{i}^{\mathrm{fac}}}] \end{array}$$

for j≥2.

The structure of the four components of the gradient, $dV/d\omega_{i}$ (Eq. **13**), $dV/dU_{i}$ (Eq. **14**), $dV/d\tau_{i}^{\mathrm{rec}}$ (Eq. **15**), $dV/d\tau_{i}^{\mathrm{fac}}$ (Eq. **16**) is similar to the original tempotron gradient in that each component consists of a sum over all input spikes $t_{i}^{j}$ that arrive prior to the time of the voltage maximum $t_{\max}$. Within the sums each spike is weighted by the postsynaptic Kernel $K(t_{\max}-t_{i}^{j})$ evaluated at the time difference between $t_{\max}$ and the input spike time $t_{i}^{j}$. In the four gradient components of the Tsodyks–Markram model each spike is additionally weighted by a specific dynamic factor: $u_{i}^{j}\;x_{i}^{j}$ for $dV/d\omega_{i}$ (Eq. **13**), $u_{i}^{j}dx_{i}^{j}/dU_{i}\;+\;x_{i}^{j}du_{i}^{j}/dU_{i}$ for $dV/dU_{i}$ (Eq. **14**), $u_{i}^{j}dx_{i}^{j}/d\tau_{i}^{\mathrm{rec}}$ for $dV/d\tau_{i}^{\mathrm{rec}}$ (Eq. **15**), and $u_{i}^{j}dx_{i}^{j}/d\tau_{i}^{\mathrm{fac}}\;+\;x_{i}^{j}du_{i}^{j}/d\tau_{i}^{\mathrm{fac}}$ for $dV/d\tau_{i}^{\mathrm{fac}}$ (Eq. **16**). In addition, the components for $dV/dU_{i}$, $dV/d\tau_{i}^{\mathrm{rec}}$, and $dV/d\tau_{i}^{\mathrm{fac}}$ are scaled with the overall synaptic scale $\omega_{i}$. The dynamic factors of the four components are shown in *SI Appendix*, Fig. S3, for an example input pattern.

For the dynamic synapse the momentum heuristic is invoked for all four synaptic parameters on any error trial where the synapse is active before $t_{\max}$, independently of whether the gradient contribution of a particular parameter is nonzero. This implementation enforces concurring updates of all four synaptic parameters, reflecting their joint role in shaping the time evolution of the instantaneous synaptic efficacies.

In the *Inset* of Fig. 2*A*, we reported the capacity of the dynamic synapse model under partial training. In these simulations, the untrained parameters were taken from an independent simulation of the same scenario in which all synaptic parameters were trained (*SI Appendix*, *Initial Synaptic Parameters*).

### Critical Capacity.

We report the storage capacity of neuron models with different types of synaptic dynamics. Due to the lack of an existing analytical calculation for the capacity of our models, we use simulation experiments to determine the critical learning load where solutions to the classification task cease to exist and convergence times approach infinity. Specifically, we estimate the critical capacity $\alpha_{c}$ as the pole of the curve

[17] $$\begin{array}{c} \Gamma \left(\alpha \right)=\frac{\Gamma_{0}}{{\left(1-\frac{\alpha }{\alpha_{c}}\right)}^{\gamma }} \end{array}$$

fitted to simulations. Here, Γ(α) refers to the expected median convergence time for a given load α and the parameters $\Gamma_{0}$ and γ denote the intercept and the exponent of the fit, respectively. The use of the median convergence time reflects the definition of critical storage capacity as the load where the probability that a solution exists is one half (1).

Specifically, to fit the divergence of convergence times, Eq. **17**, we sampled Γ(α) by sequentially increasing the learning load α until the median convergence time, $CT_{\mathrm{median}}\left(\alpha \right)$ over 1,001 independent realizations of the task reached a learning time cutoff value $L_{\max}$. To lower the overall computational costs of these simulations, this cutoff value was increased over three successive stages, i.e. $L_{\max}=10^{4}$, $10^{5}$ and $10^{6}$. Learning loads between individual evaluations of Γ(α) were increased in steps of 1% in the first two stages, and in steps of 0.5% in the third, accommodating the increasing steepness of the curve. We estimated Γ(α) through the average convergence times CT(α) over 10 independent measurements of $CT_{\mathrm{median}}\left(\alpha \right)$. The curve was fitted to the $N_{\mathrm{data}}=20$ highest consecutive values of learning loads α for which the medians of all 10 measurements remained below $L_{\max}$ (*SI Appendix*, *Curve Fitting*). Individual fits of the model and their mean absolute relative errors

$$\begin{array}{c} \varepsilon =\frac{1}{N_{\mathrm{data}}}\sum_{i=1}^{N_{\mathrm{data}}}\frac{\left|CT\left(\alpha_{i}\right)-\Gamma \left(\alpha_{i}\right)\right|}{\Gamma (\alpha_{i})}. \end{array}$$

are shown in *SI Appendix*, Figs. S6 and S7.

To evaluate the dependence of the fits on the learning time cutoff $L_{\max}$, we repeated the fitting procedure over 10 windows of 20 data points that were successively shifted by one data point toward lower loads. These analyses confirmed that our estimators for the storage capacity, i.e. $\alpha_{c}$ taken at the highest $L_{\max}$, were approached from below with shallow slopes as the simulation time cutoff increased (*SI Appendix*, Figs. S8 and S9). Consistent with the conceptual limitation that diverging learning times of an algorithms without convergence proof only represent lower bounds of an architecture’s capacity, also our measurements of the critical points represent lower bounds. Finally, the reliability of our estimator for $\alpha_{c}$ was confirmed by its small estimated relative SDs, that remained below 0.5% in all conditions (*SI Appendix*, Figs. S10 and S11).

### Electrophysiological Data Analysis.

To test the validity of the Tsodyks–Markram model we evaluated its ability to capture the short-term plasticity of biological connections that were recently made available in the Synaptic Physiology Dataset of the Allen Institute (40, 51). Specifically, we focused on 1,133 (173) connections in mice (humans) that passed the provided “quality check” (qc_pass) and were probed with the standard STP protocol consisting of a total of 12 stimulation pulses that were delivered in two groups—8 and 4 pulses at 50Hz (corresponding to interpulse intervals of 20 ms within each group)—separated by a gap of 250ms. See *SI Appendix* of ref. 40 for details on cell characterizations. For a given connection, we defined the response amplitude $A_{i}$ (i=1,2,⋯,12) for each of the 12 pulses as the median value over all available trials. We discarded 12 (1) connections of the mouse (human) data whose mean response amplitude over all 12 pulses had a sign that was inconsistent with the synapse’s inferred polarity, i.e. was negative for excitatory connections or positive for inhibitory ones. In addition, we discarded 289 (58) connections of the mouse (human) data that had fewer than 4 available trials for any of the pulses.

Following ref. 40, measures for short-term plasticity induction (${\mathrm{STP}}_{\mathrm{ind}}$) and recovery (${\mathrm{STP}}_{\mathrm{rec}}$) were defined as

[18] $$\begin{array}{c} {\mathrm{STP}}_{\mathrm{ind}}=\frac{{\bar{A}}_{6-8}-A_{1}}{A_{\max}} \end{array}$$

and

[19] $$\begin{array}{c} {\mathrm{STP}}_{\mathrm{rec}}=\frac{{\bar{A}}_{9-12}-{\bar{A}}_{1-4}}{A_{\max}}, \end{array}$$

where the averages are given by

[20] $$\begin{array}{c} {\bar{A}}_{1-4}=\frac{1}{4}\sum_{i=1}^{4}A_{i},\;{\bar{A}}_{6-8}=\frac{1}{3}\sum_{i=6}^{8}A_{i},\;{\bar{A}}_{9-12}=\frac{1}{4}\sum_{i=9}^{12}A_{i} \end{array}$$

and $A_{\max}$ denotes the maximum over all 12 response amplitudes, $A_{1},\cdots ,A_{12}$, of a given connection. Note that to simplify the comparison between the electrophysiological dataset and the Tsodyks–Markram model, we chose to normalize the differences in response amplitudes (cf. Eqs. **18** and **19**) by the maximal strength $A_{\max}$ of a given connection and not its approximation by the 90th percentile that was used in ref. 40. In Fig. 4 *A* and *F* we show the $R^{2}$ goodness of fit statistics between the biological measurements and model fits for each of the four average responses underlying the two STP behaviors, i.e. $A_{1}$, ${\bar{A}}_{1-4}$, ${\bar{A}}_{6-8}$, and ${\bar{A}}_{9-12}$ (Eq. **20**). For each connection, parameters for the Tsodyks–Markram model were obtained by running the minimize function of the scipy.optimize package to minimize the mean square error over all 12 response amplitudes $A_{1},\cdots ,A_{12}$. To alleviate the possible convergence of these searches to local minima, we reran the minimizations for each connection over the grid of all 6×7×3×3=378 possible initial guesses with w={0.01,0.05,0.5,1.0,5.0,10.0}, U={0.01,0.1,0.25,0.5,0.75,0.9,0.99}, $\tau_{\mathrm{rec}}=\left\{0.01,0.1,1.0\right\}$, and $\tau_{\mathrm{fac}}=\left\{0.01,0.1,1.0\right\}$.

In Fig. 4 *C* and *H*, we report the Kolmogorov–Smirnov test statistic for goodness of fit between the distributions that result from the multiple-spikes-per-afferent classification task ($n_{\mathrm{spikes}}$ = 4, α=6.7) and the electrophysiological data as well as the fitted Tsodyks–Markram synapses. These test statistics were evaluated through the ks_2samp function of the scipy.stats package. Tsodyks–Markram connections were separated into excitatory (52%) and inhibitory (48%) populations on the basis of the sign of w.

## Supplementary Material

Appendix 01 (PDF)

## Data Availability

Analysis and simulation codes are available on GitHub (https://github.com/guetig/pnas_2025) (62). Previously published data were used for this work (51).
