# Supplementary material for: Interactions between long- and short-term synaptic plasticity transform temporal neural representations into spatial
Source: Proc Natl Acad Sci U S A. 2025 Nov 21;122(47):e2426290122. doi: 10.1073/pnas.2426290122 (PMC12663931; doi:10.1073/pnas.2426290122)
Supplement: Supplementary file 1 — Appendix 01 (PDF) [file pnas.2426290122.sapp.pdf]

Supporting Information for

Interactions between long- and short-term synaptic plasticity transform temporal neural representations into spatial

Qiang Yu, Misha Tsodyks, Haim Sompolinsky, Dietmar Schmitz and Robert Gütig

Robert Gütig
E-mail: robert.guetig@charite.com

This PDF file includes:

Supporting text
Figs. S1 to S12

Supporting Information Text

Materials and Methods

Optimization of learning parameters. Our approach to estimate storage capacity through the measurement of convergence times relies on two assumptions: Firstly, that the convergence times of the learning algorithm reflect the size of the neuron's solution space and, secondly, that the learning algorithm converges to a solution if it exists. Since we have no formal proof for either of these assumptions our results represent only lower bounds on the storage capacity. To ensure that this bound is as close to the true storage capacity as possible, it is important, especially when comparing results across different learning dynamics, to optimize the learning parameters of each learning rule that underlie the convergence time measurements.

Here we use an iterative scheme that alternates between two steps: In the optimization step, the learning parameters are optimized through Bayesian Optimization (SI Appendix, Materials and Methods: Bayesian optimization) for a given learning load α_{opt} and a fixed $L^{\mathrm{opt}}_{\mathrm{max}} = 5000$. In this step the optimization minimizes the mean convergence time over 1001 independent runs. In the subsequent calibration step, the optimal learning parameters are then used to measure the convergence times $\Gamma(\alpha)$ for increasing learning loads $\alpha > \alpha_{\mathrm{opt}}$ following the above procedure (Materials and Methods: Critical capacity) with only $L_{\mathrm{max}} = 10^4$. The resulting curve is used to manually choose the next α_{opt}, such that the expected median convergence time $\Gamma(\alpha_{\mathrm{opt}})$ shifts towards an intermediate target range of roughly 5000 to 6000, for which we typically observed best generalization to higher learning loads $\alpha > \alpha_{\mathrm{opt}}$. The iteration is terminated when $\Gamma(\alpha_{\mathrm{opt}})$ roughly lies in the target range and subsequent improvements through further optimization become small.

Implementation details. Our simulations of the different synapse types and input scenarios relied on a standard current-based leaky integrate-and-fire neuron model (cf. Eq. 2) with N^{sim} afferents eliciting $n^{\mathrm{sim}}_{\mathrm{spikes}}$ spikes per input pattern. We used the following optimized learning parameters for each case.

1-weight scenario:

N^{sim}	100	100	100	100	100
$n^{\mathrm{sim}}_{\mathrm{spikes}}$	1	2	4	10	20
η_ω	1.285750e-03	6.895450e-04	7.214520e-04	7.680000e-04	8.485210e-04
μ_ω	9.678100e-01	9.732330e-01	9.801330e-01	9.945840e-01	9.933490e-01

2-weight scenario:

N^{sim}	200	200	200	200
$n^{\mathrm{sim}}_{\mathrm{spikes}}$	1	2	5	10
η_ω	5.783220e-04	4.476432e-04	8.485210e-04	2.687659e-04
μ_ω	9.807200e-01	9.857431e-01	9.933490e-01	9.916396e-01

n_{spikes}**-weight scenario:**

N^{sim}	100	200	400	1000	2000
$n^{\mathrm{sim}}_{\mathrm{spikes}}$	1	1	1	1	1
η_ω	1.285750e-03	5.783220e-04	2.759800e-04	9.289930e-05	4.801690e-05
μ_ω	9.678100e-01	9.807200e-01	9.896770e-01	9.964670e-01	9.982140e-01

robustness to noise with overlap:

N^{sim}	100
$n^{\mathrm{sim}}_{\mathrm{spikes}}$	4
η_ω	0.00109913
μ_ω	0.92701849

Ordinal synapse models:

For simulations of the two ordinal synapse models the N^{sim} afferents of the neuron were divided into $N^{\mathrm{sim}}/n_{\mathrm{ord}}$ subgroups. Within each of these subgroups, the spike times of all n_{ord} afferents were subject to the order constrained, i.e. fired in the same order across all input patterns.

without sign-constraint:

N^{sim}	100	200	400	1000	2000
$n^{\mathrm{sim}}_{\mathrm{spikes}}$	1	1	1	1	1
$n^{\mathrm{sim}}_{\mathrm{ord}}$	1	2	4	10	20
η_ω	9.808970e-04	8.515900e-04	5.813520e-04	6.493240e-05	1.533570e-04
μ_ω	9.637560e-01	9.810440e-01	9.894450e-01	9.951750e-01	9.973150e-01

with sign-constraint:

$N_{\mathrm{ord}}^{\mathrm{sim}}$	100	200	400	1000	2000
$n_{\mathrm{spikes}}^{\mathrm{sim}}$	1	1	1	1	1
$n_{\mathrm{ord}}^{\mathrm{sim}}$	1	2	4	10	20
η_ω	1.376440e-03	4.882459e-04	5.359481e-04	4.480545e-04	5.336161e-04
μ_ω	9.650271e-01	9.996920e-01	9.997870e-01	9.998685e-01	9.999506e-01
η_z	2.649940e-03	6.295285e+00	1.301044e+02	4.531480e-03	1.149299e-01
μ_z	9.995825e-01	8.802093e-01	9.559128e-01	9.870268e-01	9.966965e-01

Tsodyks-Markram:

fully trained:

N^{sim}	100	100	100	100	100
$n_{\mathrm{spikes}}^{\mathrm{sim}}$	1	2	4	10	20
η_ω	1.182450e-03	5.736364e-05	7.439660e-05	4.290519e-05	3.270391e-05
μ_ω	9.669692e-01	9.997383e-01	9.995642e-01	9.968705e-01	9.933878e-01
η_U	1.021539e-03	3.447968e-03	2.305646e-03	1.000000e-06	1.648876e-06
μ_U	1.000000e-06	7.800816e-06	3.606090e-03	9.999990e-01	9.999973e-01
$\eta^{\tau_{\mathrm{rec}}}$	4.225731e-02	8.229514e-03	2.667723e-03	1.701361e-05	3.648162e-06
$\mu^{\tau_{\mathrm{rec}}}$	9.997322e-01	1.808225e-04	4.416846e-03	9.978102e-01	9.994118e-01
$\eta^{\tau_{\mathrm{fac}}}$	7.817476e-01	2.221535e-03	1.313842e-03	2.168751e-03	4.992433e-04
$\mu^{\tau_{\mathrm{fac}}}$	3.755371e-06	9.003834e-01	9.169465e-01	2.376358e-01	3.894504e-04

partially trained (ω):

N^{sim}	100
$n_{\mathrm{spikes}}^{\mathrm{sim}}$	4
η_ω	8.655948e-04
μ_ω	9.794206e-01

partially trained (ω and U):

N^{sim}	100
$n_{\mathrm{spikes}}^{\mathrm{sim}}$	4
η_ω	6.016747e-05
μ_ω	9.997959e-01
η_U	2.334568e-03
μ_U	1.000000e-06

robustness to noise with overlap:

N^{sim}	100
$n_{\mathrm{spikes}}^{\mathrm{sim}}$	4
η_ω	9.33465533e-03
μ_ω	5.92698976e-01
η_U	7.13968011e-06
μ_U	9.99999000e-01
$\eta^{\tau_{\mathrm{rec}}}$	1.00000000e-06
$\mu^{\tau_{\mathrm{rec}}}$	9.99983118e-01
$\eta^{\tau_{\mathrm{fac}}}$	1.00000000e-06
$\mu^{\tau_{\mathrm{fac}}}$	9.99968997e-01

Spatio-temporal correlation task. Learning parameters for each task were optimized through grid searches.

Tsodyks-Markram:

Grid: $\eta_\omega \in \{$ 5e-3, 1e-2, 2e-2, 5e-2$\}$, $\eta_U \in \{$5e-5, 1e-4, 2e-4, 5e-4$\}$, and $\eta^{\tau_{\mathrm{rec}}} = \eta^{\tau_{\mathrm{fac}}} \in \{$5e-6, 1e-5, 2e-5, 5e-5$\}$ and all momentum coefficients set to zero.

task	pairing	long delta	short delta	xor
η_ω	5.0e-3	5.0e-2	5.0e-2	5.0e-2
η_U	5.0e-5	2.0e-4	5.0e-5	1.0e-4
$\eta^{\tau_{\mathrm{rec}}} = \eta^{\tau_{\mathrm{fac}}}$	5.0e-6	1.0e-5	5.0e-6	5.0e-6

Static:

Grid: $\eta_\omega \in \{$ 1e-4, 2e-4, 5e-4$\}$, $\mu_\omega \in \{$0.99, 0.95, 0.9, 0.5, 0.0$\}$.

task	pairing	long delta	short delta	xor
η_ω	2.0e-3	2.0e-4	5.0e-2	2.0e-4
μ_ω	0.9	0.9	0.99	0.95

Original Tsodyks-Markram model. In the original Tsodyks-Markram model(25, 30, 42), the facilitating utilization variable u_i^j of our refactored formulation is multiplied by the synaptic parameter U_i. As a result the baseline values of the dynamic factors u_i^j and x_i^j (Eq. 10) become

$$u_i^1 = U_i \quad \text{and} \quad x_i^1 = 1$$

and their iterative expressions for $j > 1$ (Eqs. 11 and 12) turn into

$$u_i^j = u_i^{j-1}(1 - U_i) \exp\left(\frac{-\Delta t_i^j}{\tau_i^{\text{fac}}}\right) + U_i,$$

and

$$x_i^j = x_i^{j-1}(1 - u_i^{j-1}) \exp\left(\frac{-\Delta t_i^j}{\tau_i^{\text{rec}}}\right) + 1 - \exp\left(\frac{-\Delta t_i^j}{\tau_i^{\text{rec}}}\right).$$

The gradient of this original Tsodyks-Markram model can be calculated analogously to the derivation of the gradient of the refactored model (Materials and Methods: Dynamic synapse).

Initial synaptic parameters. Initial synaptic parameters were drawn from Gaussian distributions with means 0.0 for ω, 0.5 for U, and 0.08 for τ^{rec} and τ^{rec} and standard deviations 10^{-3} for ω and U and 0.008 for τ^{rec} and τ^{rec}. For the sign constrained ordinal synapse the initial relative magnitudes z were set to unity. In simulations with partial training of the Tsodyks-Markram model (Fig. 2, inset), the untrained parameters were taken from independent simulations of the same scenario with full training at learning load $\alpha = 2.9$ for training of only ω and $\alpha = 4.6$ for training only ω and U. These loads correspond to the respective learning loads α_{opt} of the final step in the iterative optimization scheme for the learning parameters (SI Appendix, Materials and Methods: Optimization of learning parameters) of each scenario. The corresponding distributions of the synaptic parameters are shown in Fig. S12.

Curve fitting. Measured convergence times for increasing learning loads were fitted to the divergence (Eq. 17) by the `curve_fit()` method of the `scipy.optimize` package. All parameters were bounded to $[0, \infty)$. Initial values for Γ_0 and γ were set to 1 and the initial α_c was two times the highest learning load within the fitted data set. We used the default non-linear least square method with relative sigmas given by the standard deviation over our 10 independent measurements of the median convergence times and set the maximal number of function evaluations to 10^5. Results obtained with absolute sigma or relative sigma using the mean convergence times as scales were indistinguishable.

Bayesian optimization. For each task and synapse model, we optimized the two hyperparameters, i.e. the learning rate and the momentum parameter (Materials and Methods: Tempotron learning and Eqs. 4 and 5), that were associated with each synaptic degree of freedom. Specifically, all synapse models required the optimization of η_ω and μ_ω. In addition, the sign-constrained ordinal synapse required the optimization of η_z and μ_z, and the Tsodyks-Markram model required the optimization of η_U and μ_U, $\eta_{\tau^{\text{rec}}}$ and $\mu_{\tau^{\text{rec}}}$, and $\eta_{\tau^{\text{fac}}}$ and $\mu_{\tau^{\text{fac}}}$. We used the `skopt.gp_minimize()` function of the `scikit-optimize` package to perform simultaneous Gaussian Process optimization of all hyperparameters of a given synapse model. Learning rates were optimized on an exponential scale over six orders of magnitude in the range from 10^{-6} to 1, except for η_z with a range from 10^{-4} to 100. All momentum parameters were optimized on a logistic scale between 10^{-6} and $1 - 10^{-6}$. Specifically, we used $\eta = \exp(\hat{\eta})$ and $\mu = 1/(1 + \exp(-\hat{\mu}))$ to map the parameter values of the optimizer, $\hat{\eta}$ and $\hat{\mu}$, to the learning hyperparameters η and μ of function evaluations. Each optimization proceeded over 500 function evaluations (`"n_calls"`) including 100 random initial points (`"n_random_starts"`).

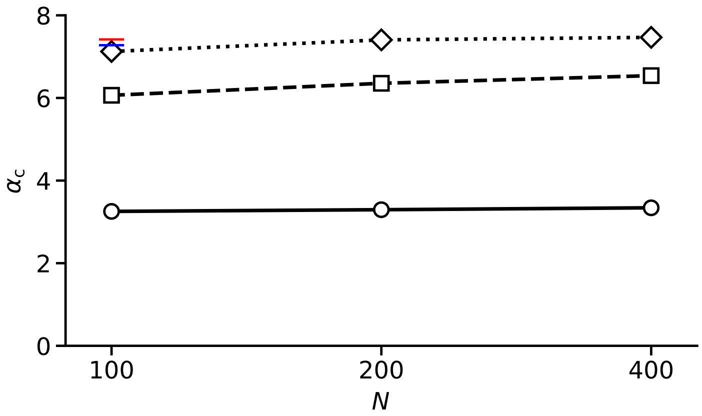

Fig. S1. Spatial scaling. Storage capacity α_c of the Tsodyks-Markram model as a function of the number of afferents N for $n_{\mathrm{spikes}} = 1$ (circles, solid line), 2 (squares, dashed line), and 4 (diamonds, dotted line) spikes per afferent. To show the saturation of the storage capacity with increasing numbers of spikes per afferent, colored horizontal markers depict the values of α_c for $N = 100$ with $n_{\mathrm{spikes}} = 10$ (blue) and $n_{\mathrm{spikes}} = 20$ (red).

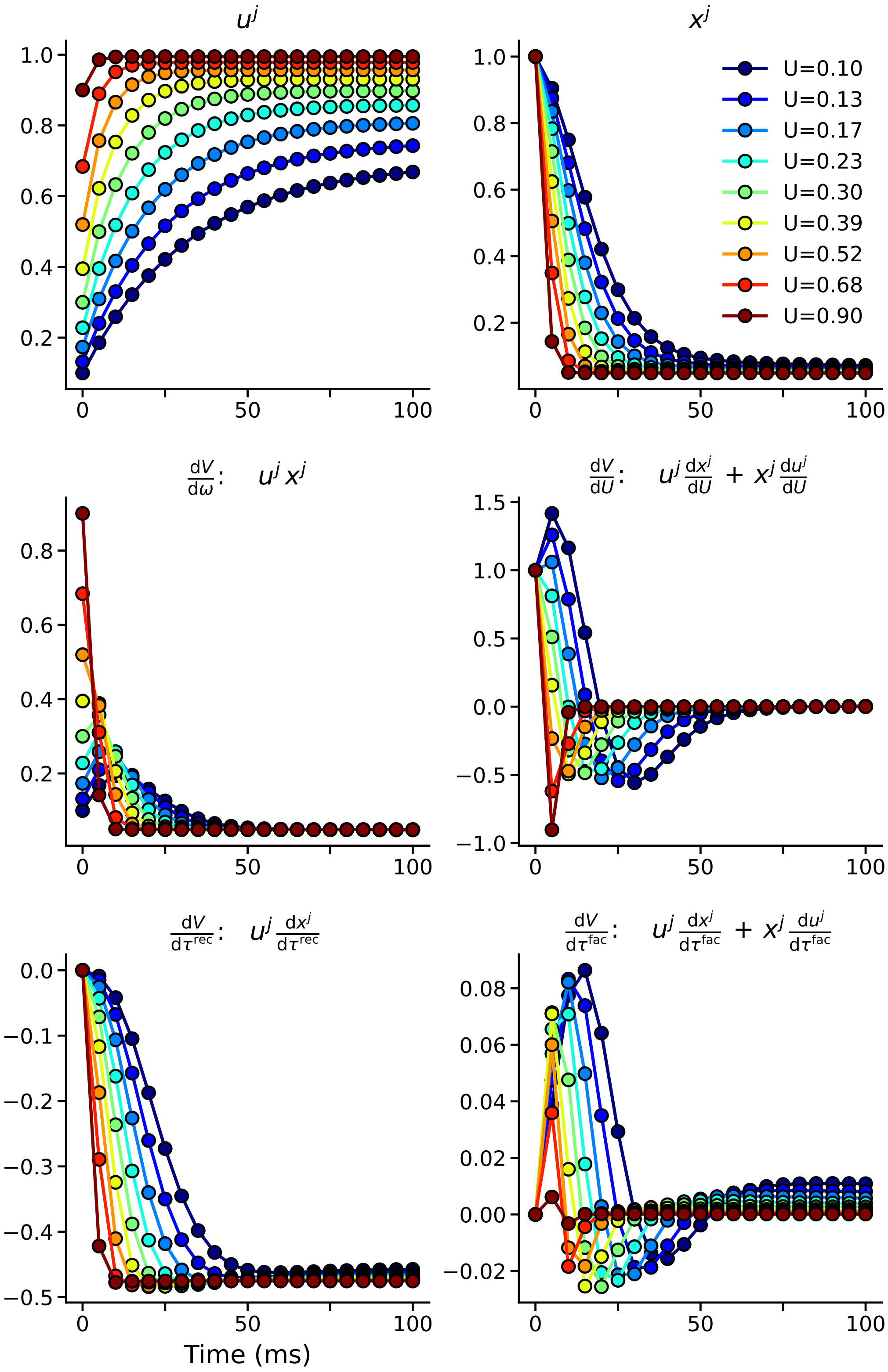

Fig. S2. Gradient components of the original Tsodyks-Markram model. Facilitating u^j (**top left**) and depressing x^j (**top right**) dynamic state variables of the original Tsodyks-Markram model over a sequence of 21 input spikes spaced 5 ms apart for different values of the utilization parameter U (**legend, top right**) and time constants $\tau_{\rm rec} = \tau_{\rm fac} = 100$ ms. The range of values for U covers facilitating (cool colors) to depressing (warm colors) synaptic dynamics. Middle and lower rows show the dynamic factors of the gradients for $\mathrm{d}V/\mathrm{d}\omega$ (**middle left**), $\mathrm{d}V/\mathrm{d}U$ (**middle right**), $\mathrm{d}V/\mathrm{d}\tau^{\rm rec}$ (**bottom left**), and $\mathrm{d}V/\mathrm{d}\tau^{\rm fac}$ (**bottom right**). See Materials and Methods for details.

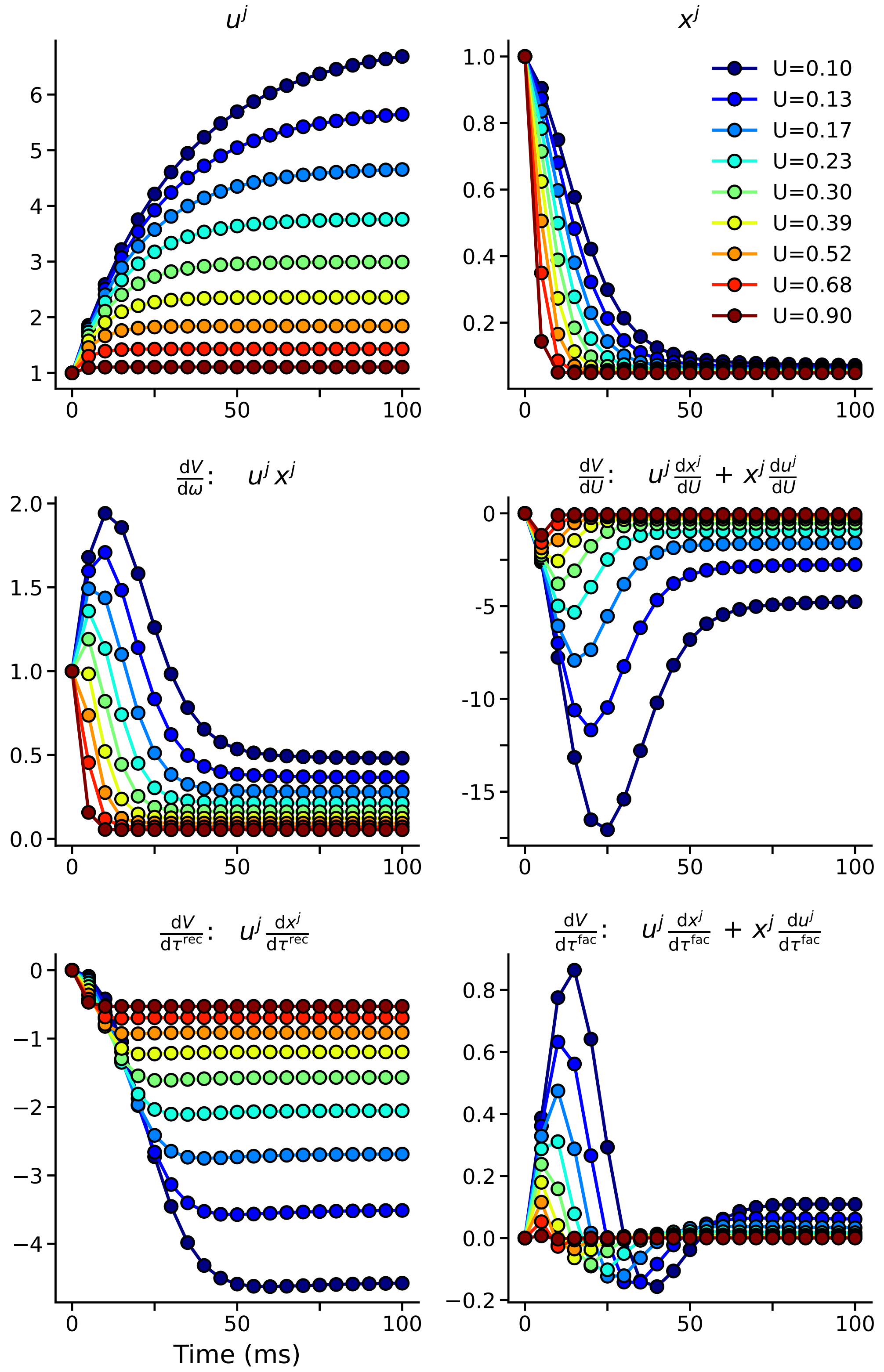

Fig. S3. Gradient components of the refactored Tsodyks-Markram model. As Fig. S2 but for the refactored Tsodyks-Markram model. See Materials and Methods for details.

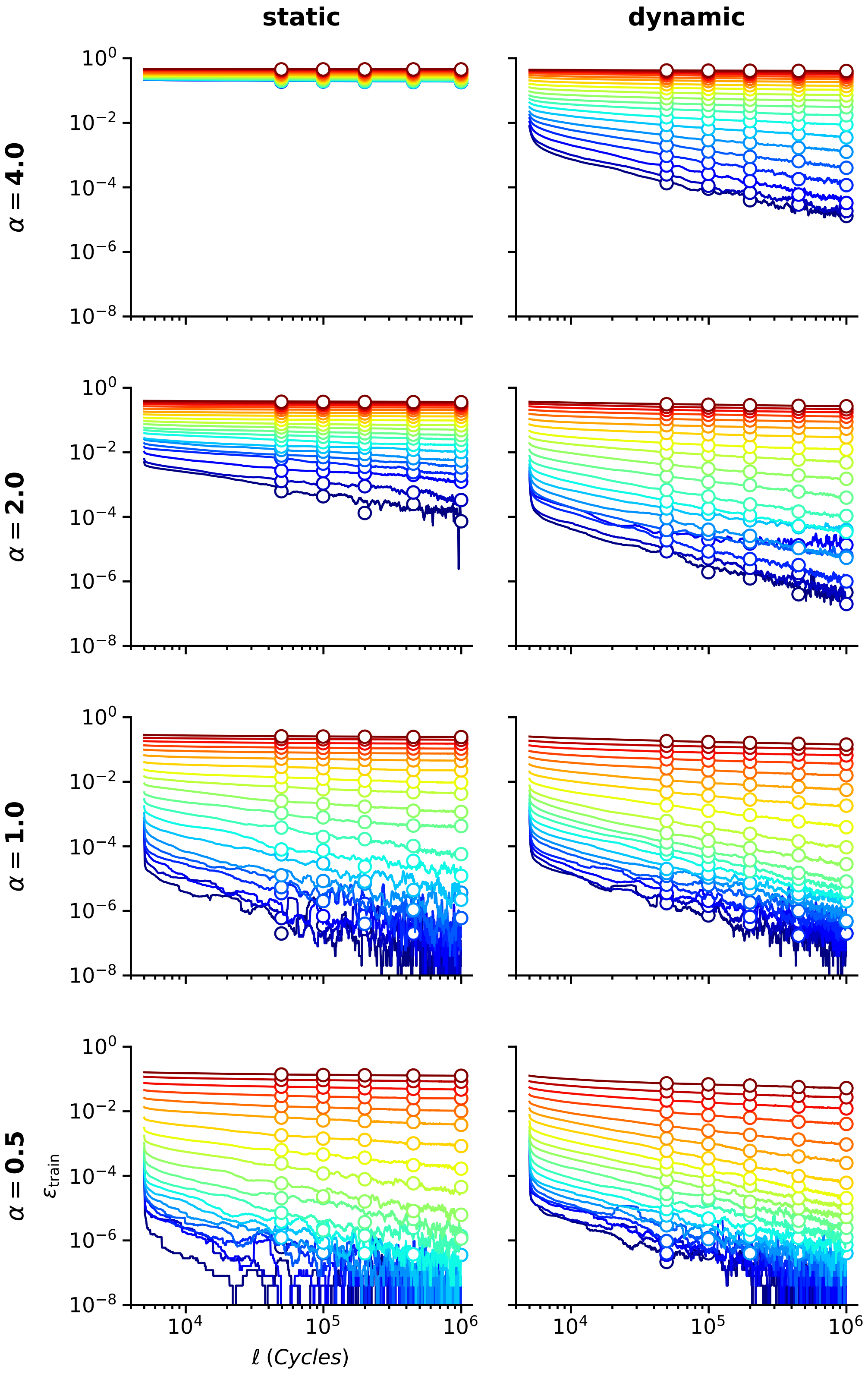

Fig. S4. Learning curves. Training errors as a function of learning time for static (**left**) and dynamic (**right**) synapse models for increasing learning loads of $\alpha = 0.5$, 1, 2, and 4 (**bottom** to **top**). Color temperatures cool (blue) to warm (red) indicate increasing levels of Gaussian spike-time jitters with zero mean and standard deviations $\sigma = 0.1$, 0.127, 0.162, 0.207, 0.264, 0.336, 0.428, 0.546, 0.695, 0.886, 1.129, 1.439, 1.833, 2.336, 2.976, 3.793, 4.833, 6.159, 7.848, and 10.0 ms. Open circles depict corresponding generalization errors (Materials and Methods) at learning times $\ell \approx 50$, 100, 200, 450 thousand and one million learning cycles.

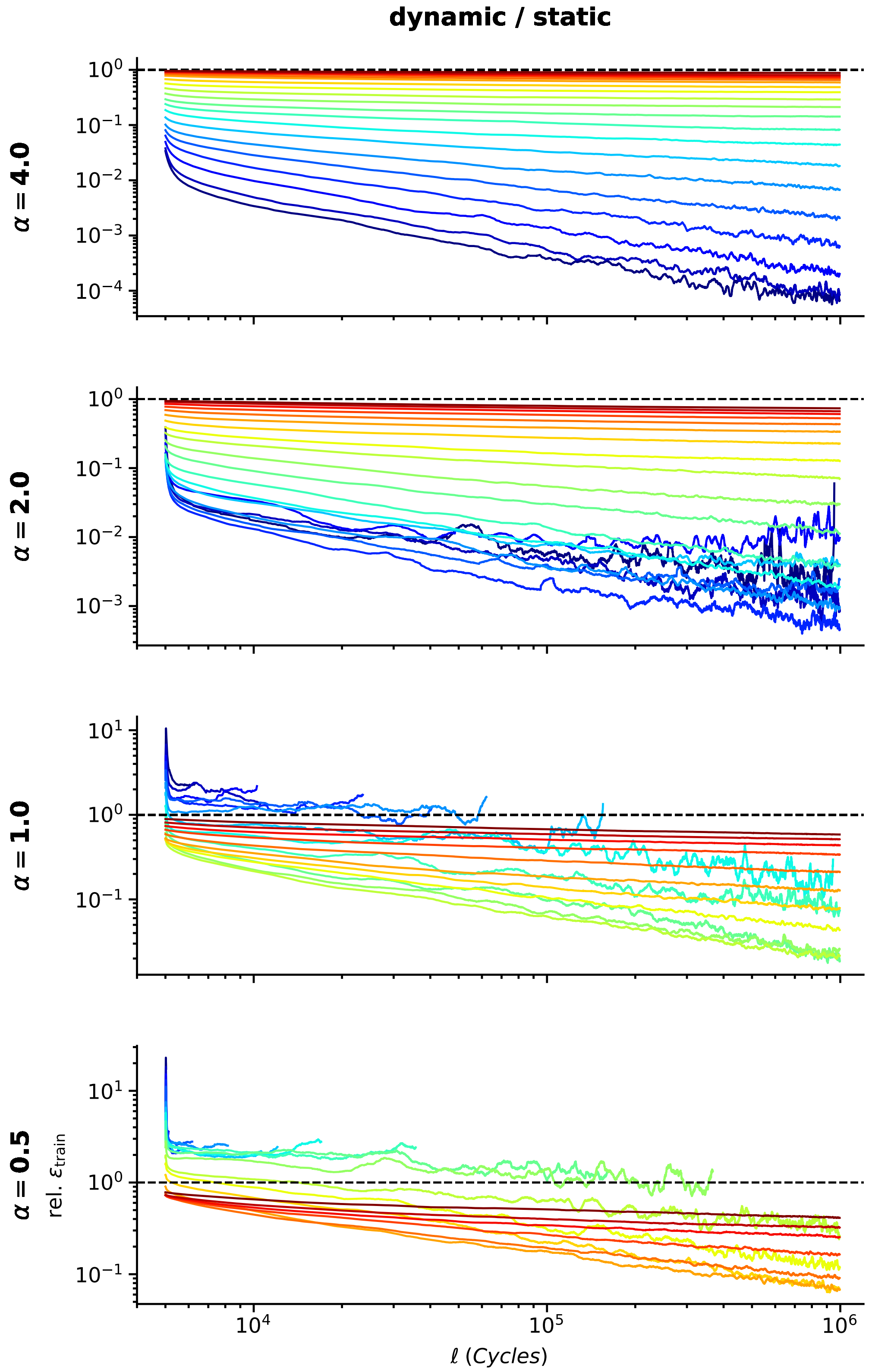

Fig. S5. Learning curve ratios. Training error ratios of the dynamic over the static synapse models as a function of learning time as in Fig. S4. Curves are terminated when the training error of the static synapse model drops below 10^{-5}.

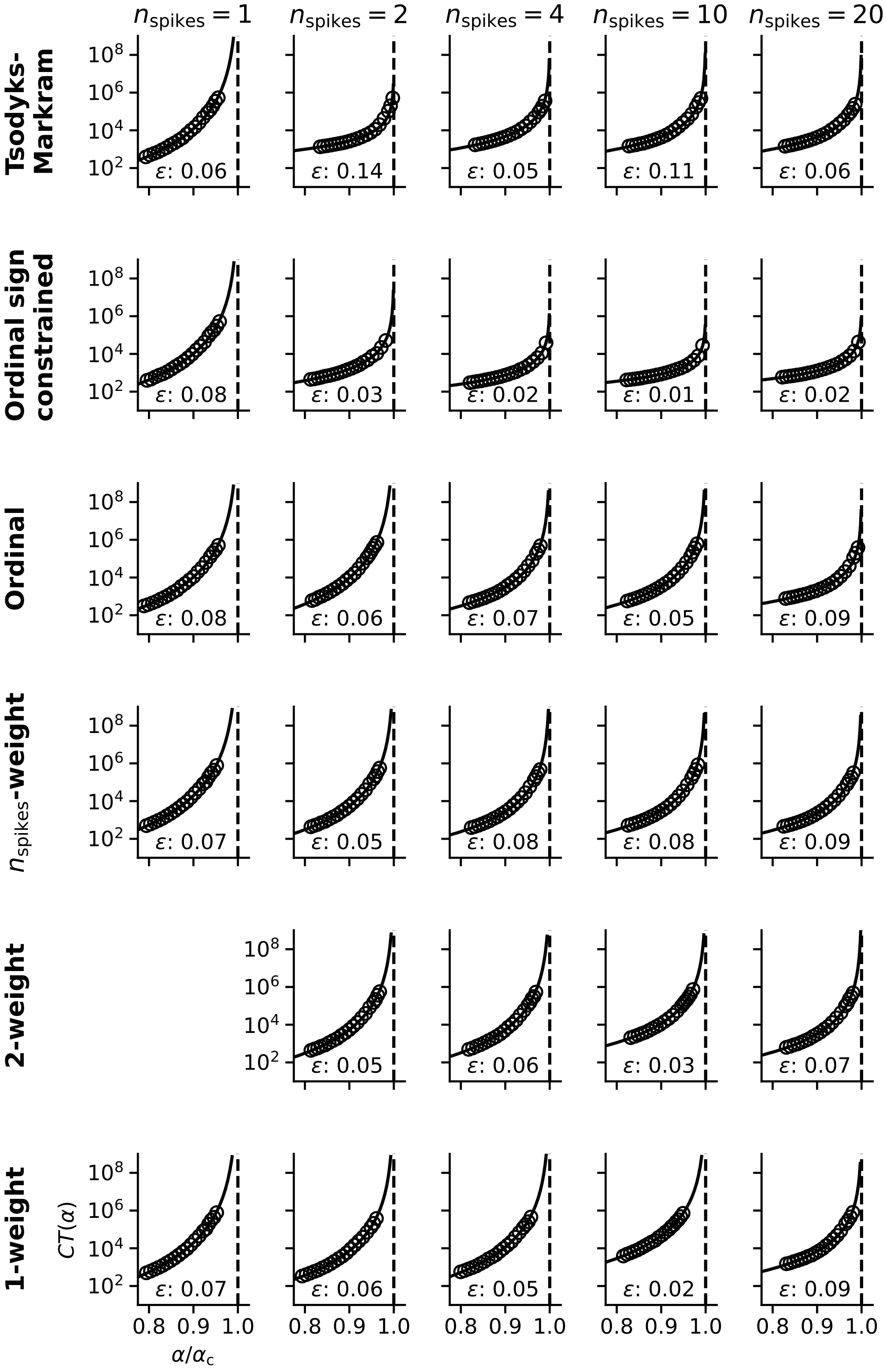

Fig. S6. Convergence time curve fits. Expected median convergence times, $CT(\alpha)$, (open circles) versus relative learning load $\alpha/\alpha_{\mathrm{c}}$ for different synapse models (rows) and different numbers of spikes per afferent n_{spikes} (columns). Solid lines depict the curve fits (Eq. 17) that underlie our estimation of the critical storage capacity α_{c} (dashed lines). The ε values given in each panel denote the mean absolute relative error of each fit. See text for details.

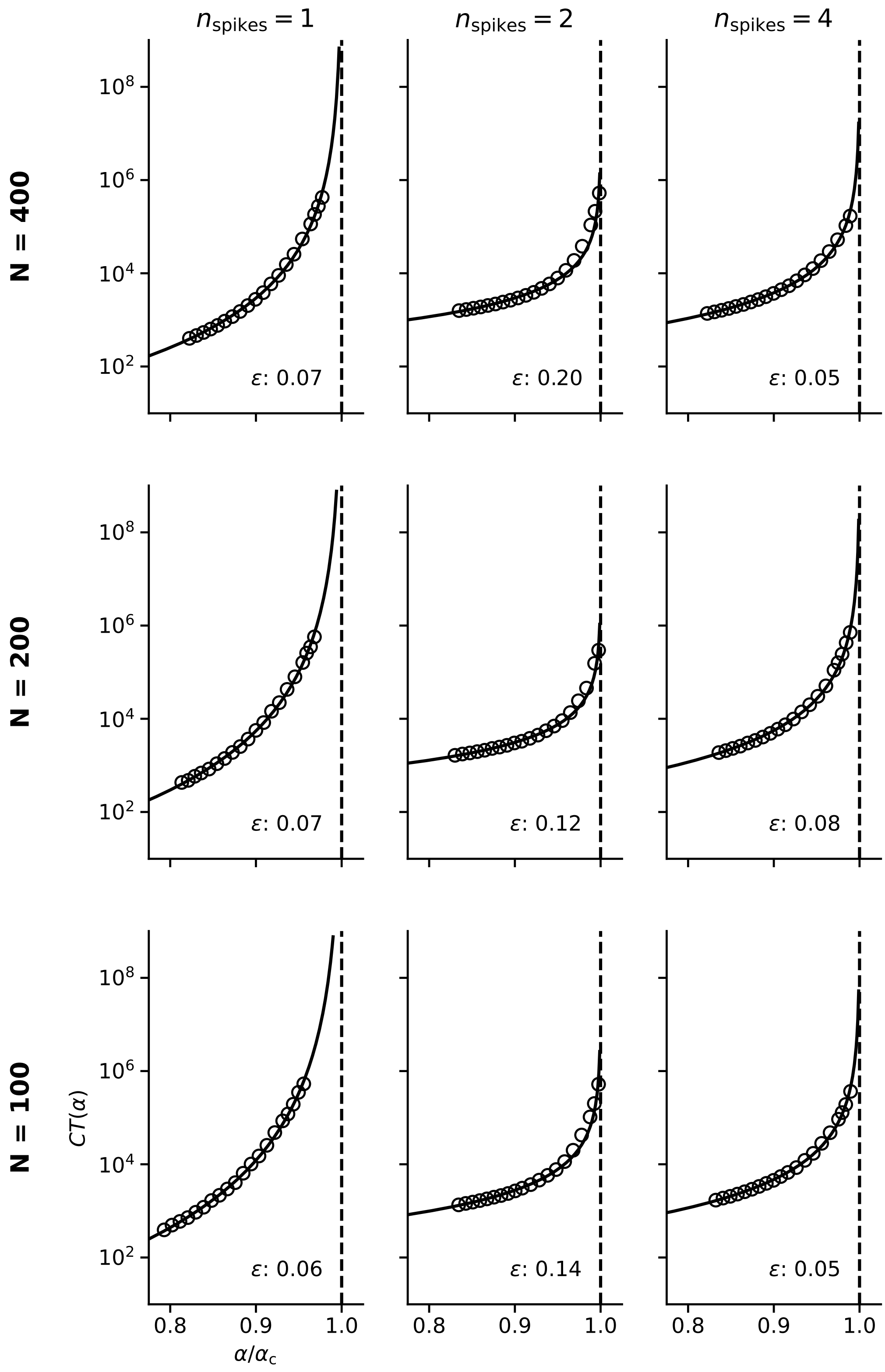

Fig. S7. Convergence time curve fits. As Fig. S6 but only for Tsodyks-Markram model with varying numbers of afferents N.

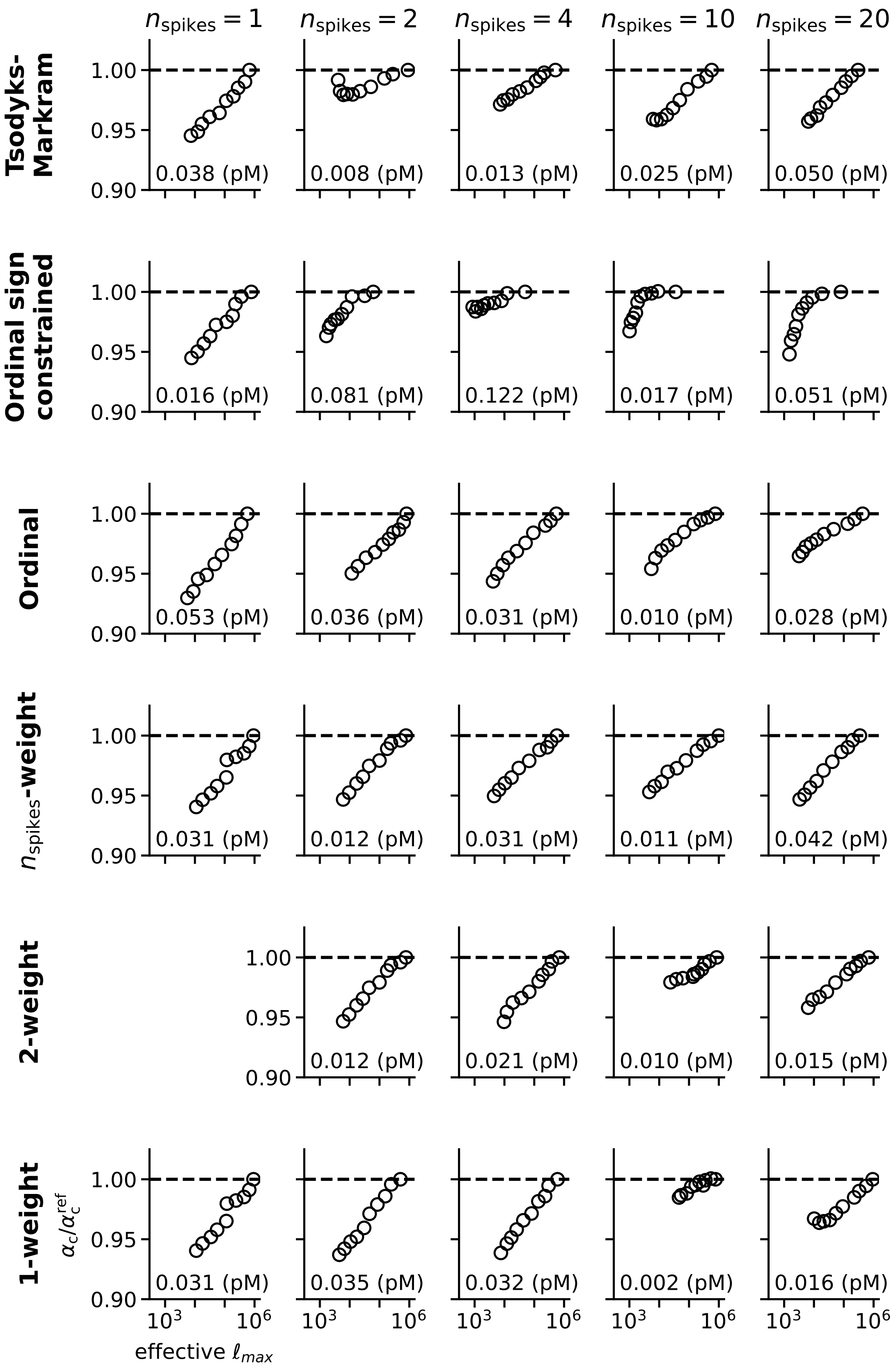

Fig. S8. Estimated storage capacity as a function of maximal learning time. Estimated storage capacities were evaluated over 10 overlapping windows of 20 data points each of which was shifted towards lower learning loads by one data point. The reference value $\alpha_{\rm c}^{\rm ref}$ (dashed lines) corresponds the storage capacity $\alpha_{\rm c}$ underlying our reported results, i.e. the value obtained by using the highest available learning loads. For each data window the effective maximal learning time $\ell_{\rm max}$ plotted on the x-axes was defined as the maximum over the 10 values of $CT_{\rm median}$ at the maximal learning load within each window. The number in each panel states the slope, per Million learning cycles (pM), of a linear regression over the last three points.

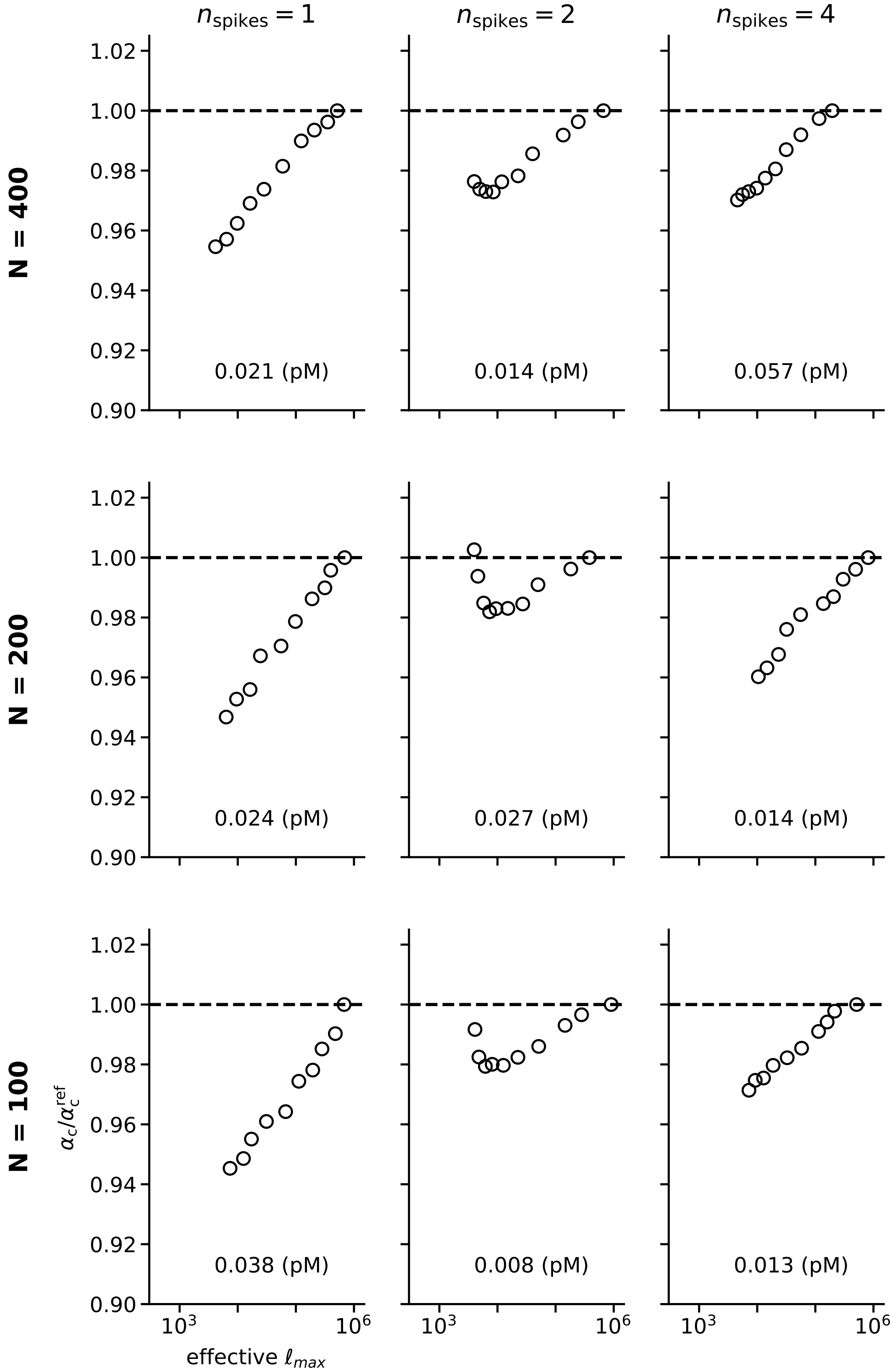

Fig. S9. Estimated storage capacity as a function of maximal learning time. As Fig. S8 but only for Tsodyks-Markram model with varying numbers of afferents N.

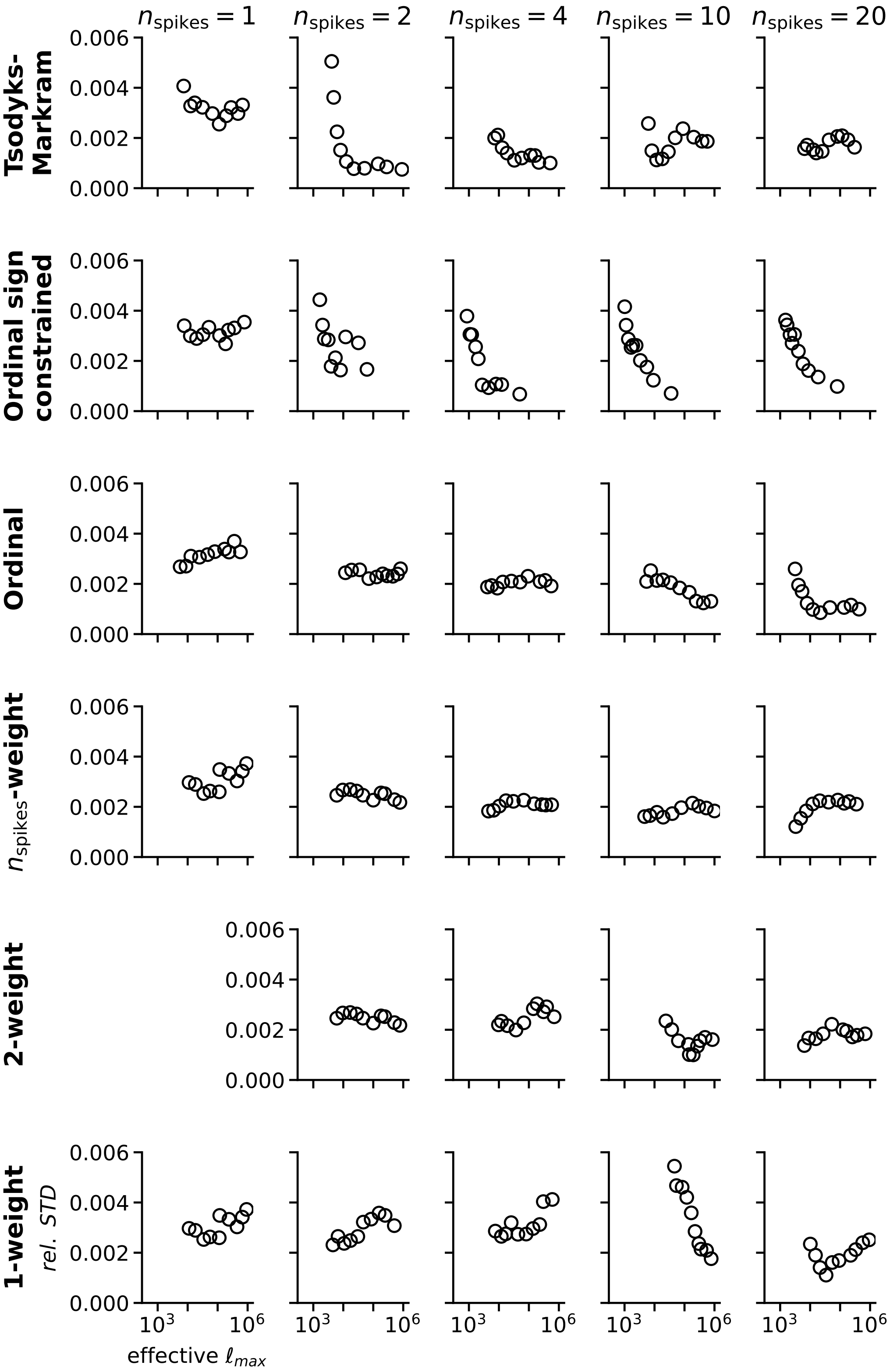

Fig. S10. Reliability of the estimator for storage capacity as a function of maximal learning time. As in Fig. S8 but showing the relative standard deviation of the estimator for α_c within each window, as estimated by the curve fitting method. See text for details.

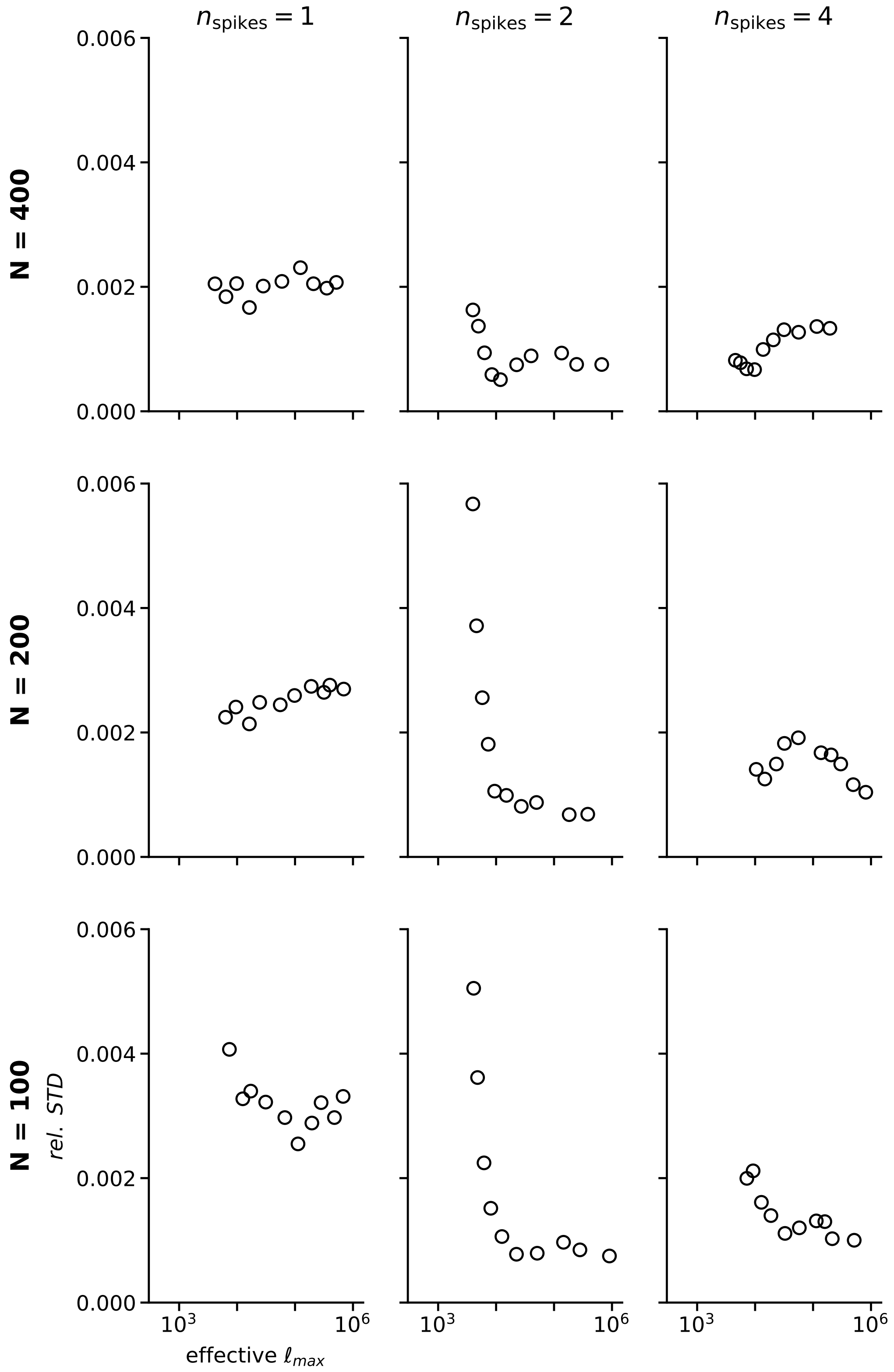

Fig. S11. Reliability of the estimator for storage capacity as a function of maximal learning time. As Fig. S10 but only for Tsodyks-Markram model with varying numbers of afferents N.

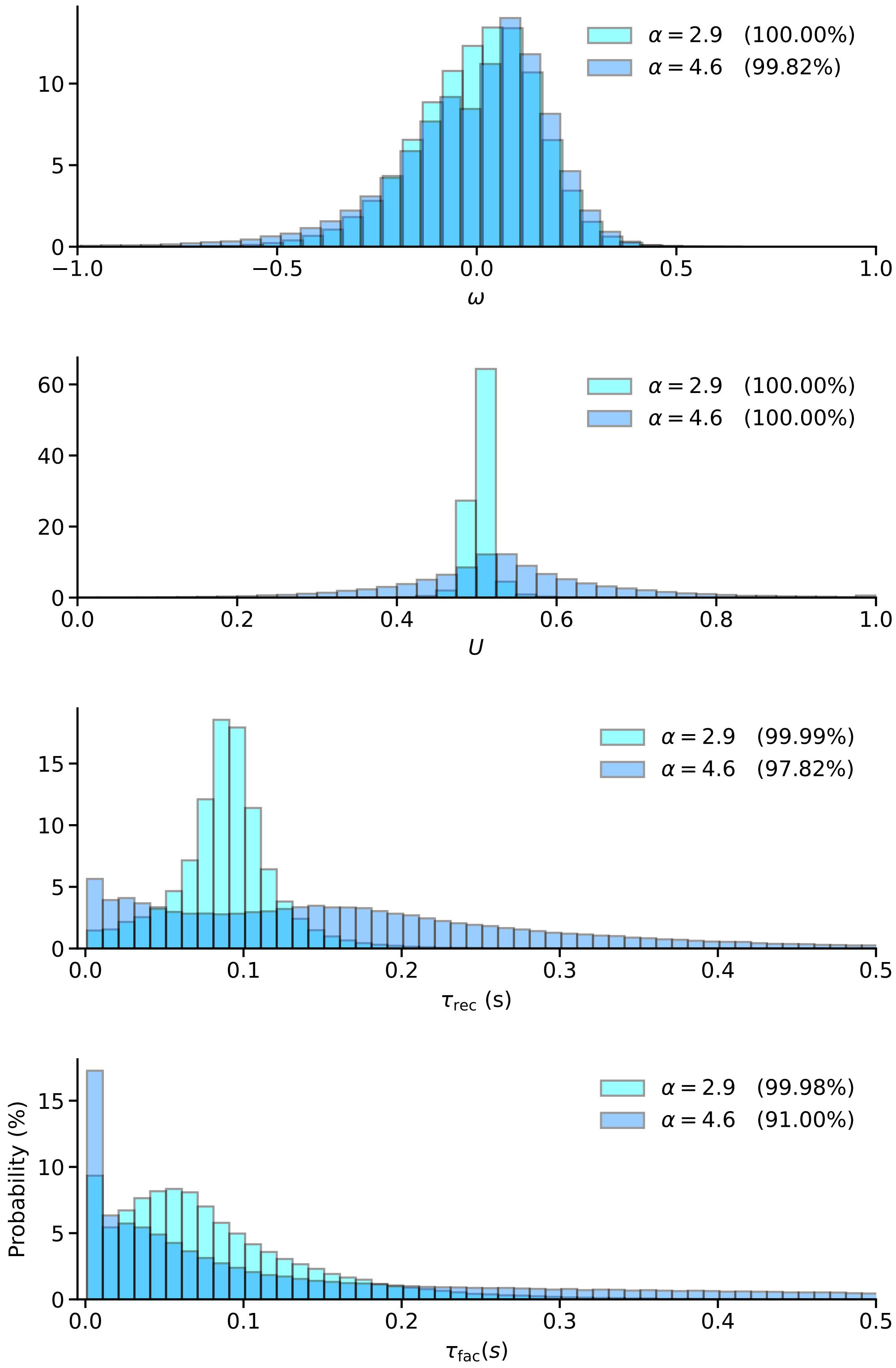

Fig. S12. Distributions of initial synaptic parameters for partial training. Synaptic parameter distributions of the fully trained Tsodyks-Markram model underlying the initialization of the partial training simulations (see text for details). Histograms were computed over 1000 independent simulations with learning loads $\alpha = 2.9$ (light blue) and $\alpha = 4.6$ (middle blue), using the same parameters as used in the initialization procedure of the partial training. Note that the limited learning time cutoff value of $L_{\max} = 10000$ resulted in 4 out of the 1000 simulations entering the histogram for $\alpha = 4.6$ to not fully converge to zero error. For better visibility, long tails of some histograms are not shown. In each row of the legends, the number in parentheses following the learning load states the accumulated probability of the shown range.
